# Telemetry-based spatial–temporal fish habitat models for fishes in an urban freshwater harbour

**DOI:** 10.1007/s10750-023-05180-z

**Published:** 2023-03-16

**Authors:** Jacob W. Brownscombe, Jonathan D. Midwood, Susan E. Doka, Steven J. Cooke

**Affiliations:** 1 Great Lakes Laboratory for Fisheries and Aquatic Sciences, Fisheries and Oceans Canada, Burlington, ON L7S 1A1 Canada; 2 Fish Ecology and Conservation Physiology Laboratory, Department of Biology and Institute of Environmental and Interdisciplinary Science, Carleton University, 1125 Colonel By Drive, Ottawa, ON K1S 5B6 Canada

**Keywords:** Acoustic telemetry, Machine learning, Great Lakes, Aquatic ecology, Fish habitat management

## Abstract

Fish habitat associations are important measures for effective aquatic habitat management, but often vary over broad spatial and temporal scales, and are therefore challenging to measure comprehensively. We used a 9-year acoustic telemetry dataset to generate spatial–temporal habitat suitability models for seven fish species in an urban freshwater harbour, Toronto Harbour, Lake Ontario. Fishes generally occupied the more natural regions of Toronto Harbour most frequently. However, each species exhibited unique habitat associations and spatial–temporal interactions in their habitat use. For example, largemouth bass exhibited the most consistent seasonal habitat use, mainly associating with shallow, sheltered embayments with high aquatic vegetation (SAV) cover. Conversely, walleye seldom occupied Toronto Harbour in summer, with the highest occupancy of shallow, low-SAV habitats in the spring, which corresponds to their spawning period. Others, such as common carp, shifted between shallow summer and deeper winter habitats. Community level spatial–temporal habitat importance estimates were also generated, which can serve as an aggregate measure for habitat management. Acoustic telemetry provides novel opportunities to generate robust spatial–temporal fish habitat models based on wild fish behaviour, which are useful for the management of fish habitat from a fish species and community perspective.

**Supplementary Information:**

The online version contains supplementary material available at 10.1007/s10750-023-05180-z.

## Introduction

Effective management of fish habitat requires consideration of the relative importance, function, and connectivity of habitat features across multiple species and life stages (Minns, 2001). These metrics often require assessment across broad spatial and temporal scales. For example, identification of spawning sites and timing is one of the most critical components of fish habitat and fisheries management (Koenig et al., 2000; Lowerre-Barbieri et al., 2021) but often requires great time and effort to effectively identify (e.g., Binder et al. 2018; Brownscombe et al. 2020b). Helping to overcome these challenges, remote tracking technologies are advancing rapidly, offering new means to characterize fish space use and habitat conditions simultaneously. In particular, acoustic telemetry, which involves tagging fish with transmitters that are tracked with specialized receivers, has become very popular for tracking fish and other aquatic organisms (Hussey et al., 2015; Matley et al., 2022). To date, much of the focus of fish acoustic telemetry has been on detecting movement to understand spatial connectivity (Brownscombe et al., 2019). Yet, there are numerous other ecological aspects that can be investigated, ranging from species-environment interactions, species-species interactions, resource ecology, and fish bioenergetics estimates, to name a few. Recently, acoustic telemetry has been used to study fish habitat use and selection more frequently (e.g., Brownscombe et al. 2021; Griffin et al. 2021; Rudolfsen et al. 2021). Although still in early development, these approaches show promise for characterizing fish space use over extensive time and space that was previously not possible. For example, habitat suitability indices can be generated through measures of habitat use or selection, identifying key habitat features that support fish populations, as well as spatial–temporal predictions of fish distributions, which may inform timing windows for anthropogenic disturbances (Brownscombe et al., 2021).

Toronto Harbour (referred to hereon as the Harbour) is situated in the central waterfront area of the City of Toronto on the north shore of Lake Ontario (43.631ºN, -79.368ºW). The Harbour serves as a hub for commercial and recreational activities for Canada’s most populous city (population of 2.93 million), and is widely developed for numerous uses with human-made (mainly concrete) shorelines and numerous marinas (Lehrer & Laidley, 2008; Doka et al., 2018; Leisti et al., 2020). In addition to altered shorelines and structured aquatic habitat, the Harbour also experiences eutrophication due to runoff from urban and agriculturally dominated watersheds (Howell et al., 2018). Although all regions of the Harbour are influenced by human activities to some degree, the outer regions of the Harbour have more natural shorelines and are generally less affected by land-use related water quality issues (Doka et al. 2018). Because of water quality impairments, loss of fish and wildlife habitats, and degradation of other biotic communities, the Toronto region (including the harbour) was designed as an Area of Concern (AOC) in 1987 under the Great Lakes Water Quality Agreement. Despite progress towards alleviating impairments related to degraded benthic communities and animal deformities, impairments still persist in water quality, fish habitat, and fish populations productivity (Midwood et al., 2021a). Indeed, recent assessments of the fish community in Toronto Harbour still indicate lower indices of biotic integrity than other similar nearshore areas (Hoyle et al., 2018; Midwood et al., 2022).

Starting in 2010, Fisheries and Oceans Canada, in collaboration with Carleton University and Toronto and Region Conservation Authority, began tracking fish space use within the Harbour. Species captured in sufficiently high abundance, and of medium to large body size (generally, > 30 cm total length) have been tagged, including largemouth bass [*Micropterus salmoides* (Lacepède, 1802)], northern pike (*Esox Lucius* Linnaeus, 1758), walleye [*Sander vitreus* (Mitchill, 1818)], yellow perch [*Perca flavescens* (Mitchill, 1814)], bowfin (*Amia calva* Linnaeus, 1766), common carp (*Cyprinus carpio* Linnaeus, 1758), and white sucker [*Catostomus commersonii* (Lacepède, 1803)]. This tracking program has generated results on the scales of fish space use across seasons and fish sizes (Midwood et al., 2019), as well as fish occupancy of artificial and restored habitats in the inner Harbour (Rous et al., 2017; Veilleux et al., 2018). Recent sampling and modelling efforts have made more fish habitat data available, including the distribution of submerged aquatic vegetation (SAV), a key fish habitat variable in nearshore freshwater systems (Midwood et al., 2021b). Combined with advances in analytical techniques (e.g., Bayesian and machine learning analyses; Hostetter and Royle 2020; Brownscombe et al., 2021), this opens new avenues of research opportunity for characterizing adult fish habitat associations by species with telemetry data.

Toronto Harbour represents an important nearshore area for both human activities and aquatic life, including fish populations, necessitating monitoring, maintenance, and enhancement and the integrity of this nearshore aquatic system that remains designated as a Great Lakes AOC (Dahmer et al., 2018; Doka et al., 2018). With extensive fish tracking data and some measures of fish habitat characteristics available on concurrent spatial and temporal scales, there is now opportunity for greater exploration of fish-habitat relationships with telemetry. Our objective was to generate spatial–temporal fish habitat models for the seven fish species with extensive acoustic telemetry fish tracking data available in this system. In doing so, we aimed to provide insights into the habitats these species use over space and time to inform habitat management (e.g., restoration; Stille et al. 2018) in the Harbour and similar species and systems. Moreover, the approach used here will have relevance to the monitoring of fish and fish habitats in other regions where the impacts of human activities on aquatic ecosystems are being managed.

## Methods

### Data collection

**Fig. 1:**
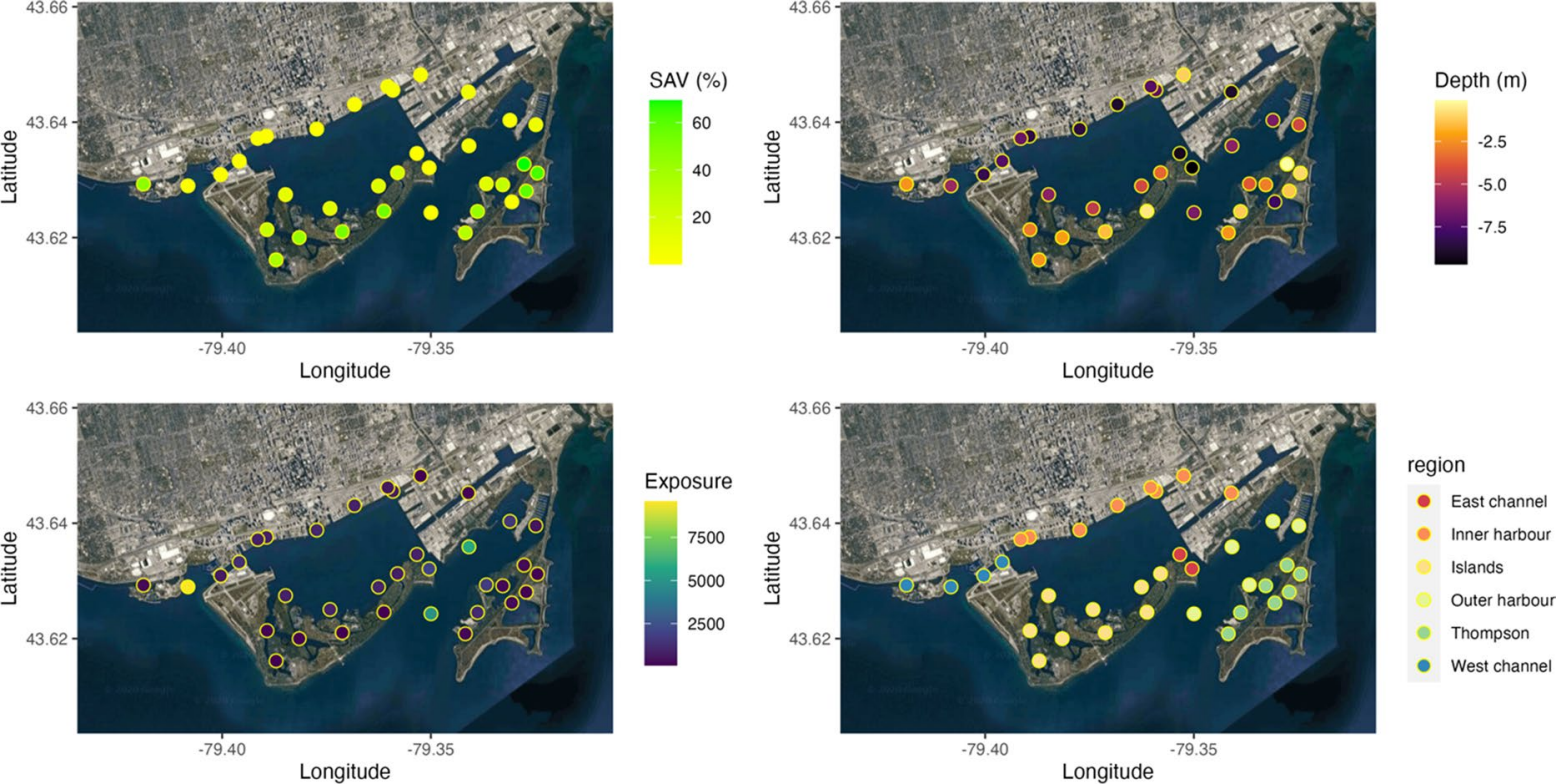
Habitat variables at acoustic receiver locations in Toronto Harbour used to generate spatial–temporal fish habitat models. SAV (%) = submerged aquatic vegetation % cover, Exposure = mean fetch (meters)

The habitat use of largemouth bass, northern pike, walleye, yellow perch, bowfin, common carp, and white sucker was measured using acoustic telemetry over a 9-year period from Sep-2010 to Oct-2019. Fish were captured by electrofishing and tagged with acoustic transmitters (Models V7-4x, V9-2x, V9TP-2x, V13-1x, V13A-1x, V13P-1x, V13TP-1x; Vemco Inc., Halifax, Nova Scotia) via surgical implantation in the coelomic cavity (see Midwood et al. [2019] for more details on fish capture and tagging). All procedures were conducted under Carleton University Animal Care Committee Application #110,723. Tagged fish were tracked with acoustic receivers (Vemco VR2W; n = 67). At each receiver, habitat variables were estimated, including the water depth (meters), percent cover of submerged aquatic vegetation (SAV), and wind exposure (Fig. 1; see Midwood et al. [2019] for more details on habitat sampling). Receivers were grouped into 36 receiver nodes that represented discrete locations with similar habitat characteristics (Appendix S1; Fig. S1). Each node was then assigned a general regional descriptor, as either Outer Harbour, Tommy Thompson Park (TTP), Toronto Islands, West channel, East Channel, or Inner Harbour (Fig. 1). Receiver deployment periods at these nodes were variable but spanned at least one year, with numerous years of coverage at multiple receiver nodes for each region of the Harbour (Appendix S1; Fig. S2). Detection ranges were measured at a subset of these receivers, and varied from 400 to 1500 m (Veilleux, 2014).

### Data analysis

All data analyses were conducted in R v1.2.5019 (R Core Team, 2019). Fish detections logged on acoustic receivers were filtered to remove any potentially false detections (Simpfendorfer et al., 2015). Detections that occurred from the same tag at the same receiver within a period of less than the minimum tag delay (45 s) were removed, as were single detections that occurred within a one-hour time period. Once filtered, reliable detections of individual tags were plotted over space and time to visually examine stationary tags (i.e. those not tracking live fish due to fish mortality or tag shedding; Klinard & Matley, 2020). Any tagged individuals that were detected repeatedly over multiple weeks at the same receiver and were not subsequently detected at any other receiver were considered to be ‘dead’ tags and these detections were filtered out of the dataset as well (Appendix S1; Figs. S3-S9).

This resulted in a total of 27.9 million detections from 456 individual fish, with variable tracking periods amongst individuals and species (Appendix S1; Table1, Fig. S10). To examine patterns of habitat use, filtered detection data were used to generate a daily presence/absence dataset for each receiver node and species for the entire study period. Individuals could have therefore been considered present at multiple receivers in a given day. To ensure a sufficient number of individuals were being tracked for each species, the data were filtered to only include days where at least 5 individuals were being actively tracked. Individuals were considered to be actively tracked during the period between when they were first detected and last detected in the tracking system, after the above-described data filtering (Appendix S1; Fig. S11).

**Table 1:** Random forests model fit metrics for seven fish species in Toronto Harbour based on model predictions onto test data

Species	Accuracy	Kappa	Accuracy Null	Accuracy p-value	Sensitivity	Specificity	Precision	NPV	F1	Balance
Largemouth Bass	0.83	0.53	0.75	< 0.001	0.60	0.91	0.68	0.87	0.64	0.75
Northern Pike	0.70	0.40	0.52	< 0.001	0.71	0.69	0.68	0.72	0.70	0.70
Walleye	0.81	0.43	0.82	0.98	0.64	0.84	0.47	0.92	0.55	0.74
Yellow Perch	0.88	0.58	0.83	< 0.001	0.70	0.91	0.63	0.94	0.66	0.80
Bowfin	0.88	0.55	0.88	0.20	0.78	0.90	0.51	0.97	0.62	0.84
Common Carp	0.74	0.43	0.69	< 0.001	0.72	0.74	0.55	0.86	0.63	0.73
White Sucker	0.83	0.54	0.75	< 0.001	0.65	0.89	0.66	0.89	0.66	0.77

Accuracy = overall proportion of correctly predicted classes; Kappa = accuracy normalized for baseline of random chance; Accuracy Null = prediction accuracy if all predictions were the dominant class, Accuracy p-value = probability that Accuracy is the same as Accuracy Null; Sensitivity = model prediction accuracy in true presences; Specificity = model prediction accuracy in true absences; Precision = predicted presence accuracy; NPV = negative predicted value (predicted absence accuracy); F1 = harmonic mean of Precision and Sensitivity, integrating positive prediction accuracy and error; Balance = accuracy amongst classes weighted equally

For each species, a random forests algorithm (RF; Breiman, 2001) was fit to species-level daily presence-absence data at each receiver node, with corresponding habitat conditions as predictors including water depth, SAV, wind exposure, and season. All predictors except season were continuous categorical, and estimated at one time period in the summer season. Habitat conditions were estimated based on their mean value within a 350 m buffer around the receiver node (constrained to areas within water only; see Midwood et al. 2019). For each species, the data were randomly split into 70% training, 15% tuning, and 15% test datasets. RF were fit to each species’ training data with 1000 trees and the default number of variables was tried at each split, the square root of the number of predictors. Models were weighted to account for unbalanced categorical response variables to balance prediction sensitivity (true presence accuracy) and recall (predicted presence accuracy) in the tuning dataset. Specifically, zero-inflated data (> 70% zeros for each species) tend to result in models that favour accuracy of absences (model prediction specificity) due to their outsized impact on overall model accuracy, so models should be weighted to achieve better balance in presences (Brownscombe et al., 2021). Presences and absences were numerically weighted using the classwt() argument in the random forests model formulation. An iterative model tuning approach was used, assigning a wide range of weight values to each class to determine the optimal tuning based on model fit to tuning data (balancing class accuracy) and the accuracy of fitted partial dependencies relative to true mean presence probabilities (Brownscombe et al., 2021). Finalized, tuned models were used to predict onto the test dataset to calculate a range of fit metrics (described in Brownscombe et al., [2021]).

Variable importance was quantified with Mean Decrease in Accuracy (MDA), which is the percent decrease in model accuracy in fitted RF trees that did not include the variable (an integral part of RF fitting; Breiman, 2001). Interaction importance was also assessed for two-way interactions between season and all other predictors using Friedman’s H-statistic (Friedman & Popescu, 2008). Spatial–temporal habitat suitability indices were derived from these models by calculating the partial dependencies (ŷ) of each predictor variable, as well as two-way interactions between season and all other predictor variables. Partial dependencies reflect the relationship between each variable (or combination of variables with interactions) with other predictors held constant at their mean. RF were fit with the ‘randomForests’ package (Liaw & Wiener, 2002), model fit metrics were calculated with the ‘caret’ package (Kuhn et al., 2019), variable interaction values were calculated with the ‘iml’ package (Molnar, 2019), and partial dependencies (ŷ) were calculated with the ‘pdp’ package (Greenwell, 2017). Aggregate, community-level habitat importance (based on telemetry-derived habitat use for tracked species) was generated by calculating the mean partial dependencies for each two-way interaction between season and habitat variables, SAV, water depth, and region. Exposure was not assessed at this level due to insufficient data available amongst species across the range of exposure values measured in the system.

## Results

**Fig. 2:**
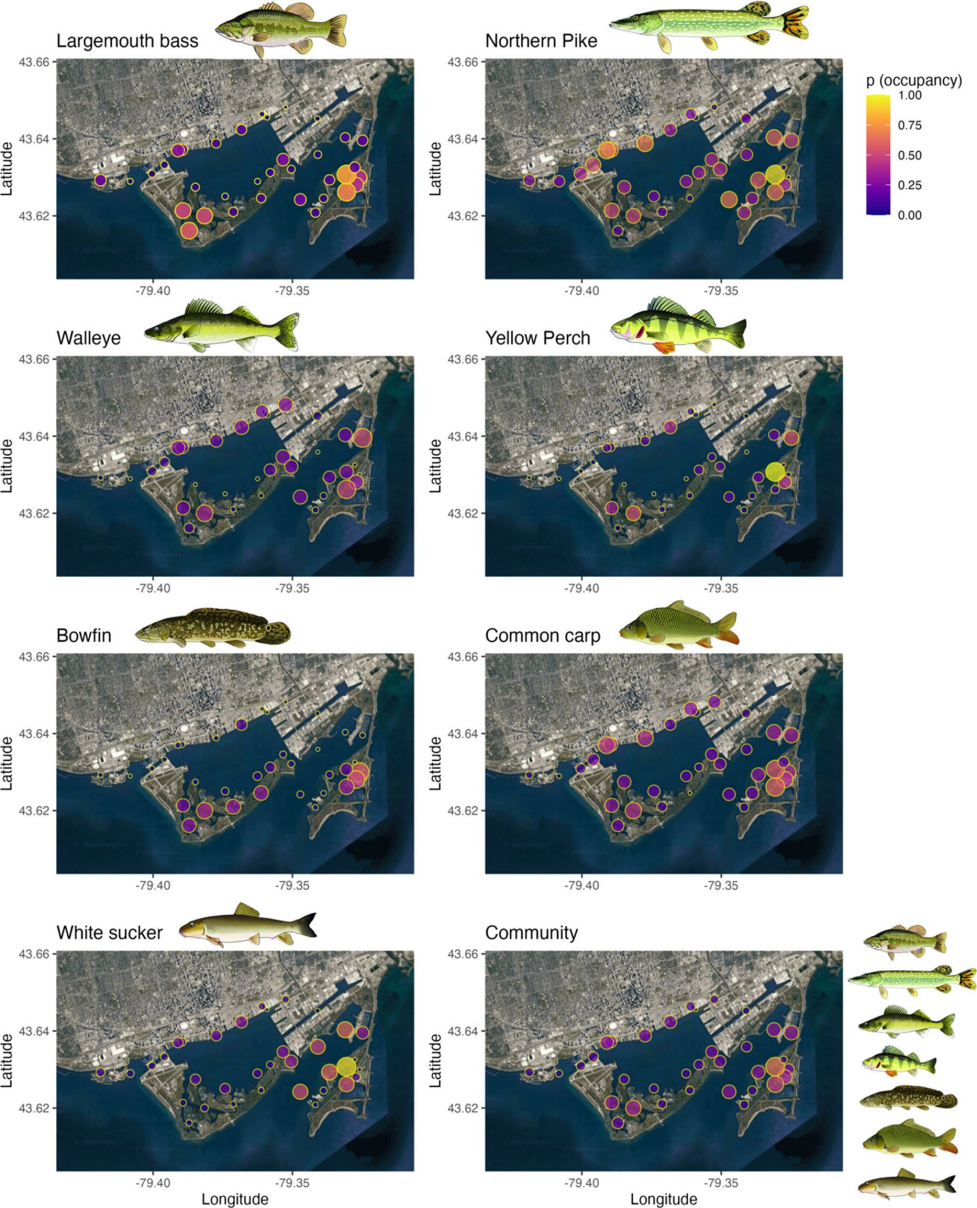
Spatial distributions of fish occupancy (probability of occurrence from presence-absence data over the course of the species-specific tracking period) for seven fish species tracked with acoustic telemetry in the Toronto Harbour area

The seven fish species tracked with acoustic telemetry in Toronto Harbour exhibited variable overall patterns in space and habitat use, with most species occupying Tommy Thompson Park (TTP), the Outer Harbour, and the Toronto Islands regions most frequently (Fig. 2). Examining spatial–temporal fish habitat use, RF models had a range of accuracy values (Table 1) in predicting the presence/absence amongst species (0.70–0.88), as well as variable sensitivity (true presence accuracy; 0.60–0.78), specificity (true absence accuracy; 0.69–0.91), precision (predicted presence accuracy; 0.47–0.68), and negative predicted accuracy (0.72–0.97).

**Fig. 3:**
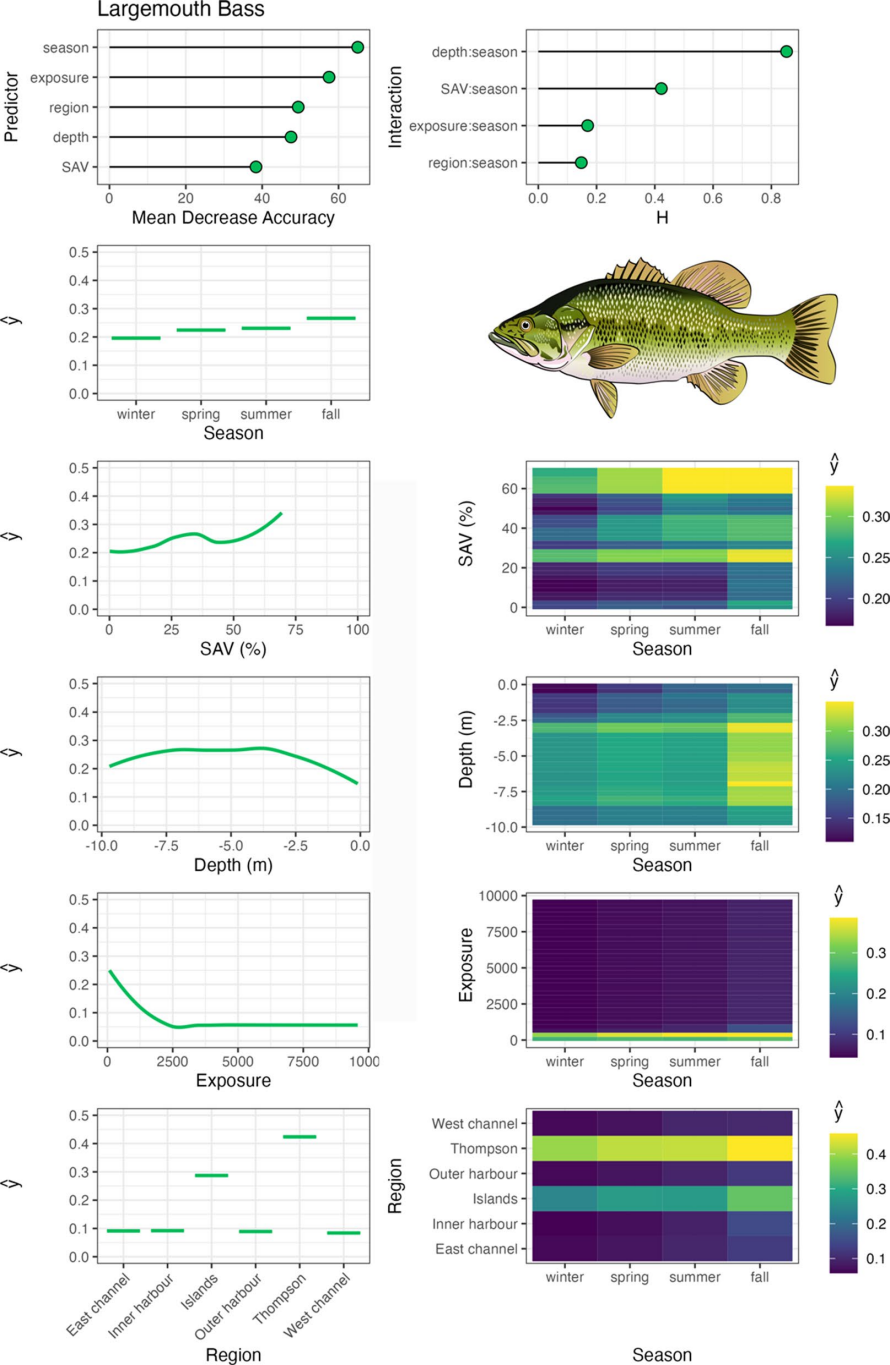
Largemouth bass spatial–temporal habitat model outputs in Toronto Harbour. Partial dependencies (ŷ) indicate the marginal effect of the predictor levels on fish occupancy

**Fig. 4:**
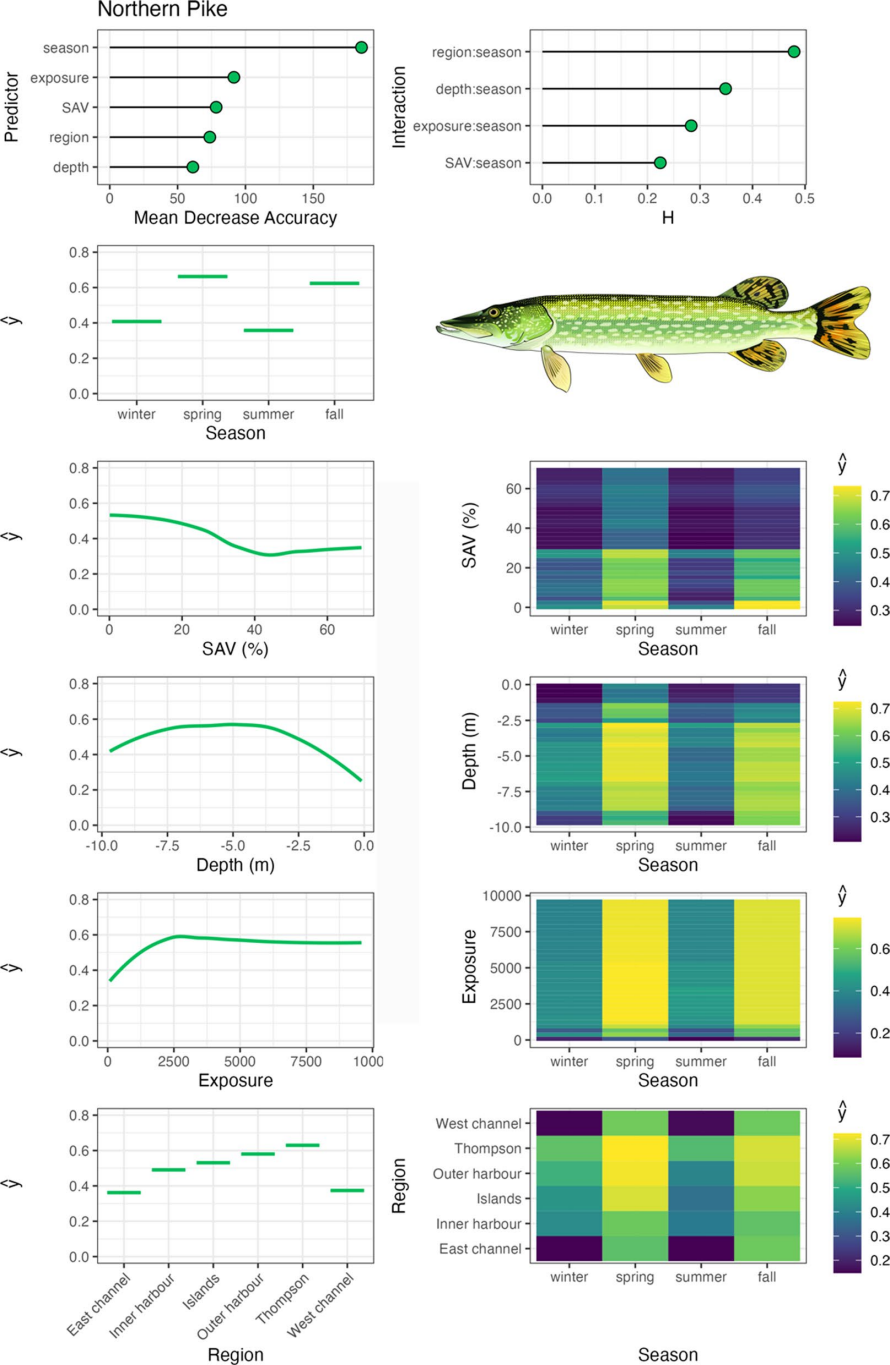
Northern pike spatial–temporal habitat model outputs in Toronto Harbour. Partial dependencies (ŷ) indicate the marginal effect of the predictor levels on fish occupancy

Largemouth bass were associated with moderate to high SAV densities, particularly with > 50% SAV densities across all seasons (Fig. 3). They occupied a wide range of water depths, with peak distribution within the 3 to 7 m range. Largemouth bass were consistently associated with sheltered, low wind exposure environments, where SAV tends to dominate in TTP and Toronto Island regions, and exhibited little seasonality in habitat use across the measured variables. In contrast, northern pike occupied numerous regions and a wide range of water depths in the Harbour, most frequently in the spring and summer months, and were associated with low SAV densities (Fig. 4).

**Fig. 5:**
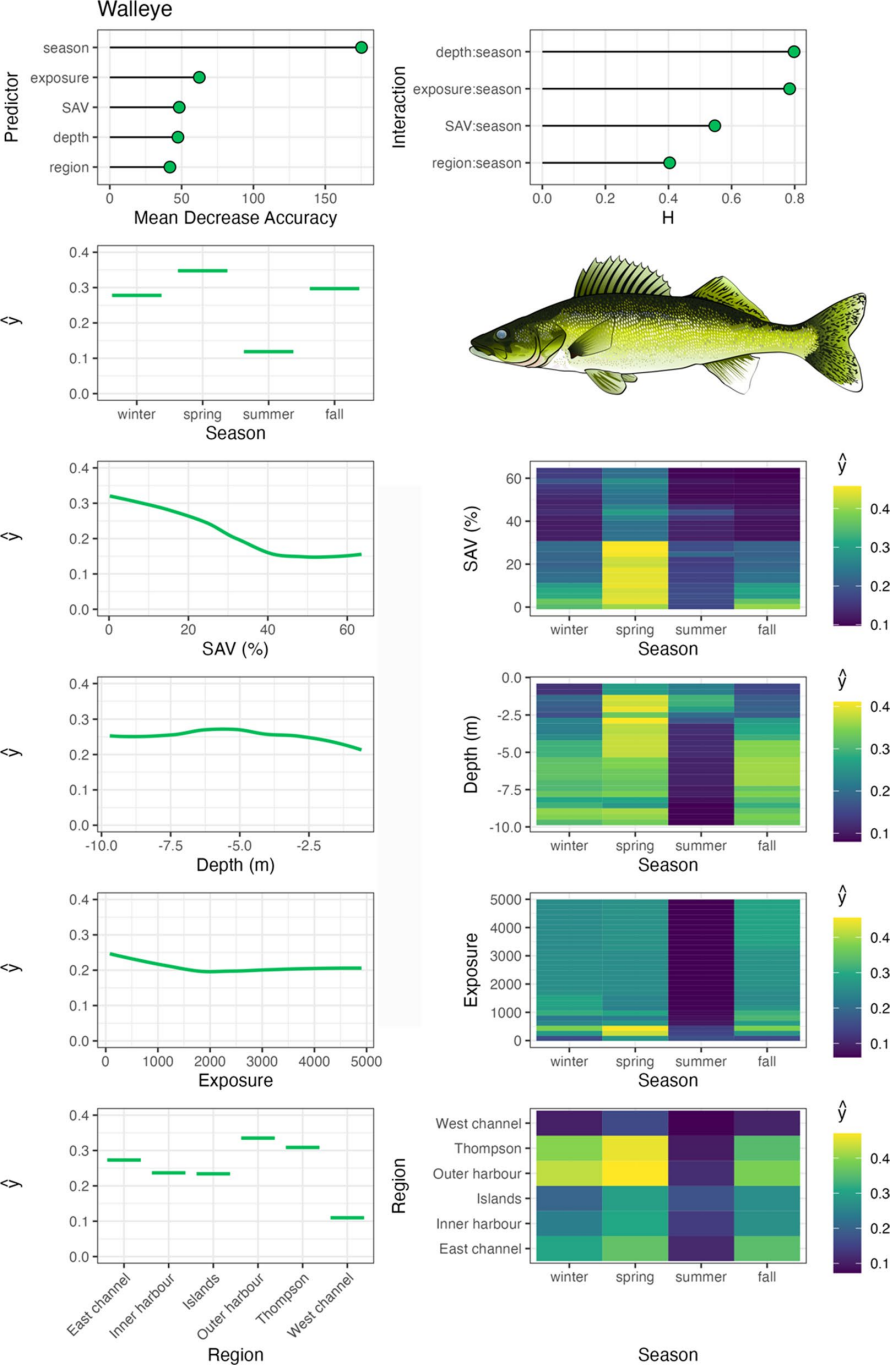
Walleye spatial–temporal habitat model outputs in Toronto Harbour. Partial dependencies (ŷ) indicate the marginal effect of the predictor levels on fish occupancy

**Fig. 6:**
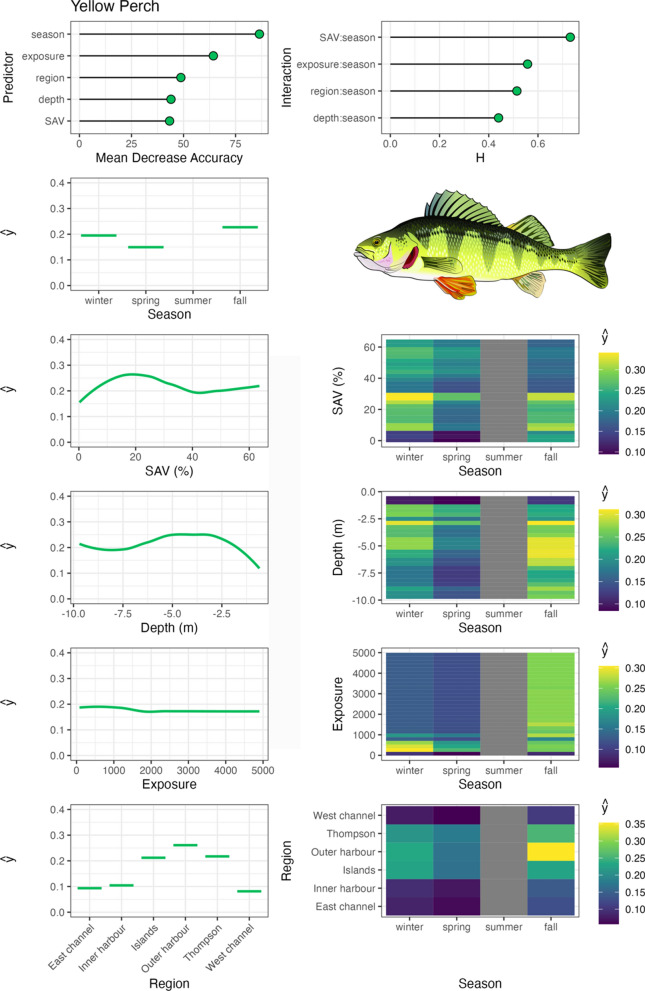
Yellow perch spatial–temporal habitat model outputs in Toronto Harbour. Partial dependencies (ŷ) indicate the marginal effect of the predictor levels on fish occupancy

Walleye occupied Toronto Harbour most frequently outside the summer, especially in the spring, during which occupancy was highest in shallow water depths with low SAV in the TTP and Outer Harbour regions (Fig. 5). In the fall and winter, walleye occupied a wide range of water depths, with a stronger association with moderate to deep waters (4–10 m). Similar to northern pike, walleye tended to occupy low-SAV densities in general and were wide ranging amongst regions. The other Percidae species, yellow perch, were also wide ranging amongst regions, also with the highest association with TTP and Outer Harbour (Fig. 6). Insufficient data were available to assess yellow perch summer habitat use; however, in other seasons they occupied a wide range of water depths and SAV densities, associating with a range of exposures in the fall, with their range contracting to low exposure habitats in the winter.

**Fig. 7:**
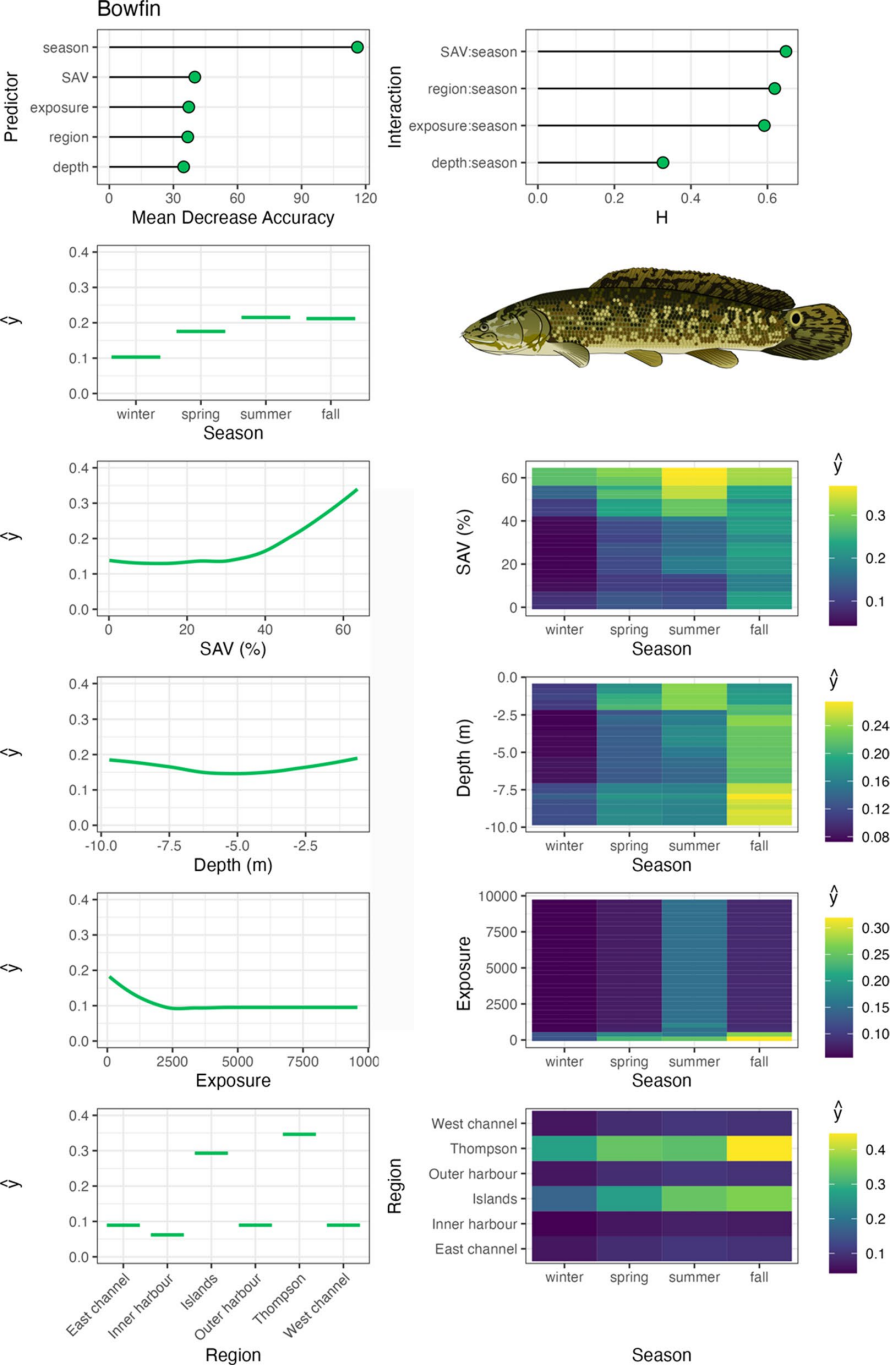
Bowfin spatial–temporal habitat model outputs in Toronto Harbour. Partial dependencies (ŷ) indicate the marginal effect of the predictor levels on fish occupancy

**Fig. 8:**
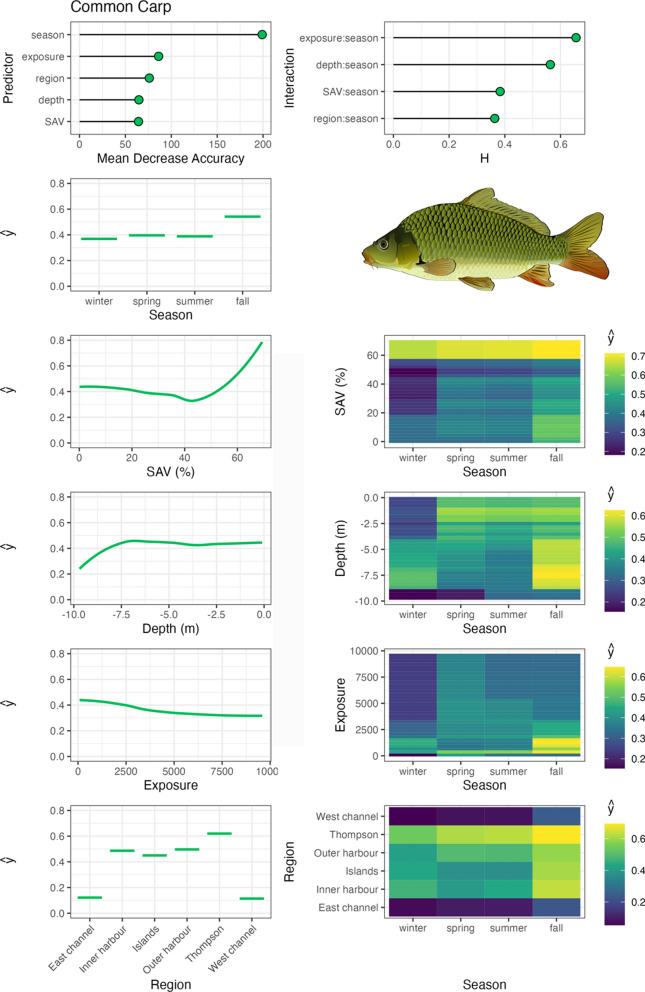
Common carp spatial–temporal habitat model outputs in Toronto Harbour. Partial dependencies (ŷ) indicate the marginal effect of the predictor levels on fish occupancy

**Fig. 9:**
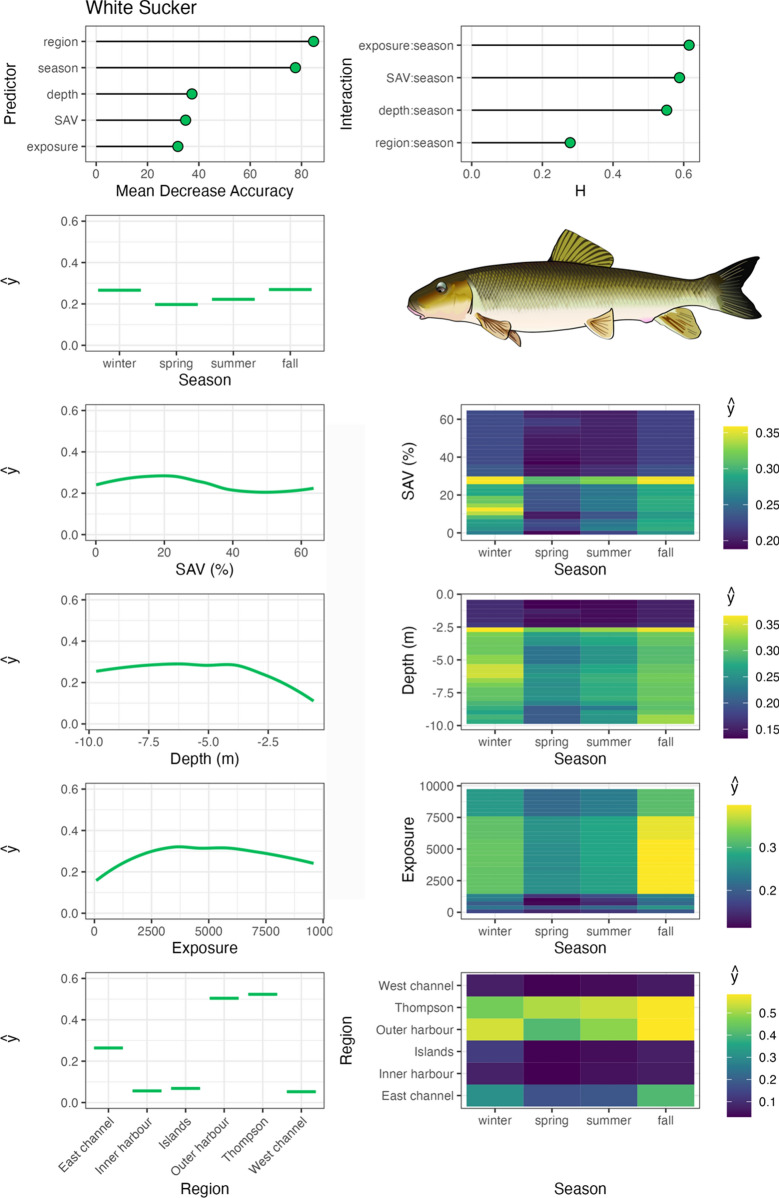
White sucker spatial–temporal habitat model outputs in Toronto Harbour. Partial dependencies (ŷ) indicate the marginal effect of the predictor levels on fish occupancy

Bowfin exhibited similar habitat associations to largemouth bass, with the highest occupancy in TTP and Toronto Islands regions in low exposure, high SAV habitats (Fig. 7). Bowfin primarily occupied shallow water depths, but transitioned into deep water in fall, and were seldom detected in the tracking system in winter. Common carp also showed similar habitat associations to bowfin and largemouth bass, with their highest occupancy of sheltered, low exposure habitats and moderate-high SAV densities (Fig. 8). However, common carp were more wide ranging, occupying TTP, Outer Harbour, Inner Harbour, and Toronto Islands regions. They exhibited a clear pattern of shallow water depth occupation in the spring and summer, transitioning in the fall to deeper water and remaining there through the winter in a range of Harbour regions. White sucker primarily occupied TTP and Outer Harbour regions, including a wide range of water depths and wind exposure values, but generally associated with low SAV densities (Fig. 9). They were detected in the tracking system less frequently in the spring and summer months.

**Fig. 10:**
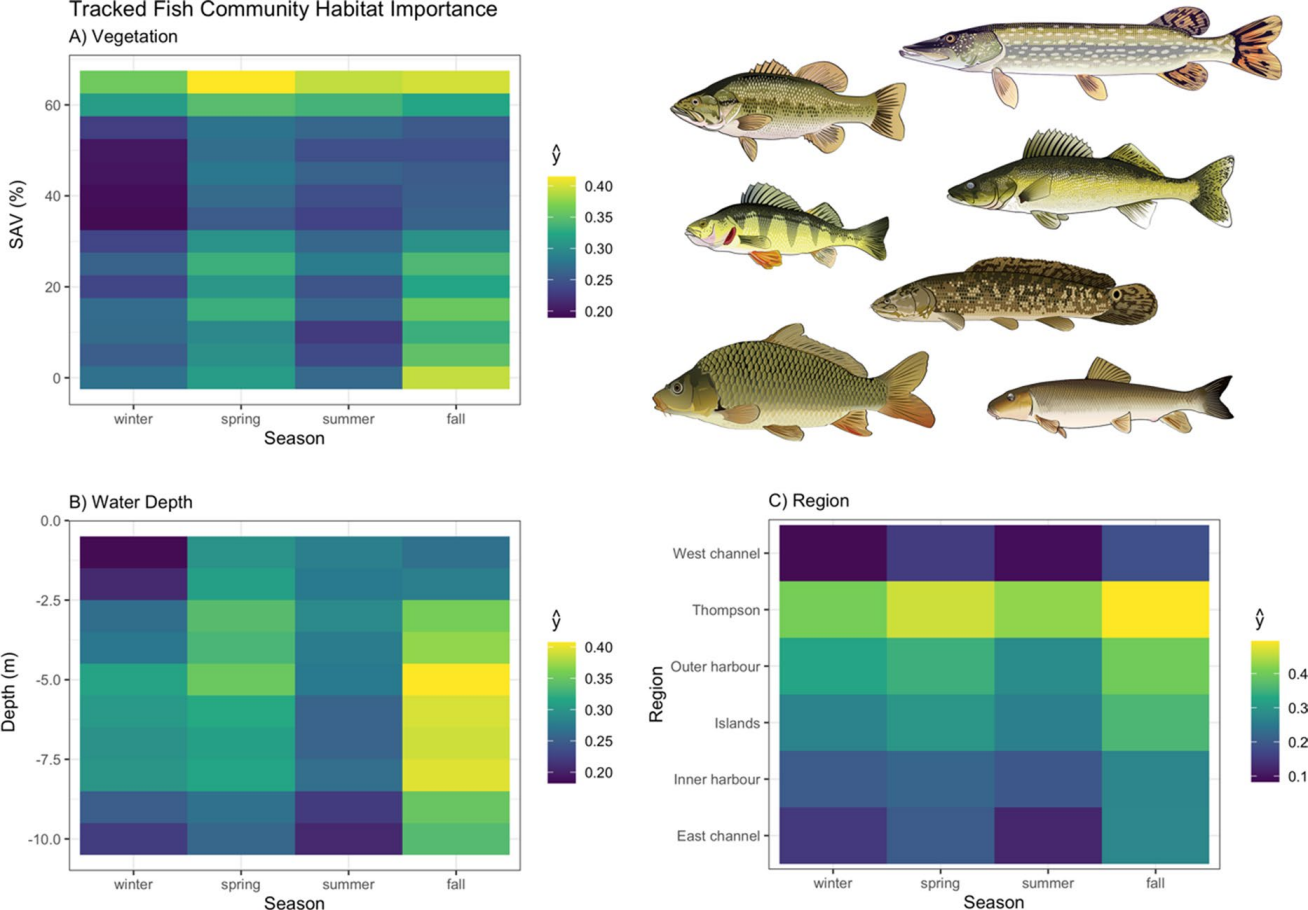
Spatial–temporal habitat importance indicated by marginal effects (ŷ) averaged from all random forests models from fish species tracked in Toronto Harbour, by season and **A** submerged aquatic vegetation (SAV; %), **B** water depth (meters), and **C** harbour region

Aggregate community-level habitat associations indicated that both high (> 50%) and moderate-low (< 35%) SAV cover are of the highest importance for the tracked species, with high SAV cover particularly important in the spring, and low-SAV cover in the fall (Figs. 2,10). Moderate–shallow water depths (< 6 m) were most important in the spring, and moderate–deep depths (4–8 m) in the fall. Amongst regions, TTP was clearly the most important habitat for these fish at the community level throughout the year, followed by the Outer Harbour and Toronto Islands (Fig. 10).

## Discussion

The spatial–temporal fish habitat models generated here from an extensive 10-year telemetry dataset provide ecological knowledge of these seven fish species, especially in the Toronto Harbour area. The fish species showed a range of spatial–temporal patterns in habitat use from largemouth bass, which exhibited little seasonal variation and associated consistently with sheltered, moderate-high SAV habitats, to walleye and northern pike, which showed more intermittent occupancy of the Harbour and higher seasonal variation in habitat use. Aside from largemouth bass, most species exhibited high seasonal variation in habitat associations, highlighting the need for a variety of connected habitat types to sustain fish populations, likely including connectivity between the Harbour and the main body of Lake Ontario.

Prior to further interpretation of the habitat associations developed here, it is important to consider the nature of these data. Acoustic telemetry provides comprehensive spatial–temporal monitoring of fish space use, but does have caveats. In particular, acoustic telemetry via fixed receiver stations continuously monitors specific locations, but habitat use outside these locations is unknown (but in some cases can be roughly estimated; Brownscombe et al. 2019). This is especially relevant when there are large spatial gaps in detection coverage within a system, which was the case here, specifically in open, deep-water regions of Toronto Harbour (Fig. 1). Therefore, fish use of this habitat type may be generally underrepresented, and this may be the cause of lower occupancy rates within the study area for most fish species during winter.

Similarly, in the Toronto Harbour dataset, receiver coverage was more limited in very shallow water (< 2.5 m). Further compounding this issue, detection range, and therefore fish detectability, is often significantly lower in very shallow water (Kessel et al., 2014). Shallow areas are also commonly vegetated (if sheltered), and aquatic vegetation reduces detection substantially (Weinz et al., 2021). The importance of shallow and high SAV cover habitats, both combined and independently, are likely underrepresented in these outputs. Indeed, Midwood et al. (2018, 2019) surmised based on movement patterns that species such as largemouth bass and bowfin are unlikely to be exiting the Harbour and instead had evaded detection in shallow areas with dense vegetation coverage. To generate robust estimates of habitat use/selection, there must be extensive coverage across a wide range of each ecological variable being considered. Further discussion of logistical and analytical considerations for overcoming these caveats is included below.

Overall, the patterns of habitat use by the seven fish species examined here are generally consistent with their known ecology. Largemouth bass associate with sheltered, low exposure habitats and moderate-high densities SAV throughout the year, which is well established as essential foraging and spawning habitat for this species (Miranda & Pugh, 1997; Cudmore-Vokey & Minns, 2002). Notably, due to data availability, a single measure of SAV cover in the summer at each receiver (Midwood et al., 2019) was used in the model, so any positive or negative associations in other seasons may not be related to the presence of live SAV at the time, but rather other characteristics of these habitats (e.g., mud substrate, biodegrading vegetation) or the conditions that can support SAV (e.g., shallow depths, soft substrate, low exposure; Midwood et al., 2021b). Similar to largemouth bass, bowfin also associated with high SAV densities in sheltered embayments in the spring and summer, but conversely, transitioned to deeper water in the fall and were seldom detected in winter, either indicating occupation of shallow and/or deep areas with no receiver coverage (Midwood et al., 2018). However, shallow, high SAV areas are important components of bowfin habitat, including for spring spawning and summer rearing and foraging, so a reasonable assumption would be staging in shallows (Cudmore-Vokey & Minns, 2002).

Northern pike are also known to associate with SAV throughout their life history with adults preferring moderate SAV densities (Cudmore-Vokey & Minns, 2002), although individuals within a population can show considerable variation in habitat selection (Kobler et al., 2009). In Toronto Harbour, they associated more with low-moderate SAV densities, with the highest detected occupancy in the spring and fall in the outer harbour regions (TTP, Outer Harbour, Toronto Islands), where they were detected at relatively lower frequencies in the summer and winter. This occupancy pattern may result from variation in behavioural types of northern pike (Kobler et al., 2009), with some individuals exhibiting seasonal migrations in and out of the Inner Harbour. Northern pike spawn in shallow vegetated areas in the spring (Cudmore-Vokey & Minns, 2002), a habitat use pattern observed in Toronto Harbour in outer harbour areas and near the West Channel (Veilleux et al., 2018). Low summer occupancy of the Harbour is perhaps surprising. Temperatures in the Harbour do commonly exceed their temperature preferendum (20ºC; Cudmore-Vokey and Minns, 2002; Hlevca et al. 2015); however, they do not exceed their upper incipient lethal temperature (31ºC; Hlevca et al., 2015). It is possible that detection issues within the tracking system decreased apparent occupancy during these seasons. Northern pike that shifted to deeper and cooler waters with more limited receiver coverage and those that adopted an ambush foraging strategy in shallow vegetated areas (Kobler et al., 2009) would have fewer detections and thus appear absent within the array. They are capable, however, of more extensive movements (Pierce, 2013) and use of summer and winter habitats outside of Toronto Harbour by a portion of the population is thus a possibility and should be explored using an expanded telemetry array. Such an expansion of space use for this species is particularly important to determine given recent findings of declines in abundance of this critical apex predator (Midwood et al., 2022).

Walleye were seldomly detected in Toronto Harbour in the summer, but were present frequently in all other seasons, especially in the Outer Harbour. Walleye spawning occurs in the spring on rocky substrate in water currents or rocky lacustrine reefs (McMahon et al., 1984; Jennings et al., 1996). Thus their occupancy of shallow, low-SAV habitats in the Harbour in the spring may be spawning related. Walleye are tolerant of a broad range of environmental conditions, but are generally more successful in low light conditions, moderate turbidity, cool (< 24ºC) water temperatures and sufficiently high (> 5 mg/L O_2_) dissolved oxygen (McMahon et al., 1984; references therein). Walleye are known to be a wide-ranging species with large home ranges in the Great Lakes (Hayden et al., 2014; Vandergoot & Brenden, 2014). They commonly occupy similar thermal habitats to Toronto Harbour throughout the year (Madenjian et al., 2018); yet, avoidance of > 20ºC has been hypothesized as a driver of walleye movement in Lake Erie (Raby et al., 2018). Walleye are known to associate with moderate structural cover, including SAV (McMahon et al., 1984), but rarely occupied SAV habitat in Toronto Harbour. Overall, these patterns suggest that the Harbour was not summer habitat for this species; however, study design constraints limit our ability to determine why walleye appear to seldomly use Toronto Harbour in the summer. Given evidence of summer occupancy by walleye in a nearby system with more severe water quality (i.e., hypolimnetic hypoxia in Hamilton Harbour; Brooks et al. 2019), this pattern is unlikely to be driven solely by water quality and may rather be a natural component of walleye ecology in Lake Ontario (as observed in the eastern portion of the Lake; Elliot et al. 2021). The other member of the Percidae family tracked here, yellow perch, had insufficient data available to assess summer habitat use in Toronto Harbour, but occupied a wide range of exposures, water depths, but was most often found at moderate SAV densities in the Outer Harbour, TTP, and Toronto Islands regions in fall and winter. Yellow perch have been documented to spawn in the spring in shallow nearshore, vegetated areas (Krieger et al., 1983), consistent with their occupancy of moderate SAV cover and shallow areas of Toronto Harbour in the spring.

Common carp occupied a wide range of habitat types in our study, with the highest occupancy of sheltered high SAV habitats in the spring and summer in TTP and the outer Harbour, which is likely foraging and spawning habitat (Cudmore-Vokey & Minns, 2002). Common carp transitioned from these spring/summer habitats to deep water (> 7 m) habitat use in the fall and winter, which is consistent with previous studies (García-Berthou, 2001). White sucker were present in TTP and outer Harbour regions throughout the year, peaking in the fall and winter. They occupied a wide range of habitats in moderate-deep water, moderate-low SAV densities, and a wide range of exposure habitats. White sucker are known for a moderate association with SAV as adults, but spawning typically occurs in the spring over gravel substrates in lotic systems (Cudmore-Vokey & Minns, 2002), an ecotype not captured by the tracking system, but river occupancy during the spring may indicate subsequent movement into tributaries around Toronto Harbour such as the Don River, as posited by Midwood et al. (2019).

As discussed previously, interpretation of the above findings requires consideration of the study methods. This is especially true because, although the application of telemetry to generate habitat models is not new (e.g., Rogers & White, 2007; Johnson et al. 2008; Aarts et al. 2008), it is has rarely been applied to aquatic acoustic telemetry in this way until recently (e.g., Brownscombe et al. 2021; Rudolfsen et al. 2021). Like most acoustic receiver systems, the one used in this study in Toronto Harbour was designed to track larger scale fish movements, as opposed to habitat selection. This is obvious from the tracking system arrangement, including gates of receivers and strategic placement at movement chokepoints (Appendix S1; Fig. S1,2). One clear effect of this caveat is that certain habitats are underrepresented; for example, open deep-water basins within the Harbour may be overwinter habitats for some species. Further, use of shallow and highly vegetated habitats is likely underrepresented because of a lack of detection coverage (due to limited receiver deployments and low detection efficiency). Based on the known species’ ecology and more detailed assessments of individual movement patterns (e.g., Midwood et al. 2018, 2019), low detections in winter are likely indicative of occupancy of areas with poor receiver coverage and/or detection efficiency, including deep-water basins and very shallow water areas within the Harbour for some species (i.e., largemouth bass, common carp, bowfin). Out-migration may also play some role in these observed patterns, especially with more wide-ranging predatory species (i.e., northern pike, walleye). Certainly, some combination of both of these space use patterns is possible for all species. Yellow perch and white sucker (and to some extent largemouth bass) overwintered within the harbour in habitats with stronger receiver coverage, enabling identification of habitat associations in this season.

In assessing fish spatial–temporal habitat associations, we used a somewhat cursory set of habitat measures that were available at spatial and temporal scales required to match telemetry data, relative to the suite of measures that could be generated. Future studies may consider assessing a broader range of habitat metrics (at scales consistent with the telemetry data) such as substrate types, water temperature, dissolved oxygen, water clarity / light levels, and water flow, to name a few. It would also be beneficial to survey SAV more frequently, to gain a better understanding of its variability in seasonal habitat use by fishes. Future telemetry studies focused on generating fish habitat models may also consider a more systematic acoustic receiver spatial arrangement, in a random (common in habitat studies) or grid (common in fish movement studies; Kraus et al., 2018) arrangement. It is also important to consider the scale at which habitat is measured relative to that of fish positioning from acoustic telemetry. Here a general estimate of acoustic receiver detection range was used to estimate habitat conditions (see Midwood et al., 2019 for details), and a single integrated value was used to represent each variable. In reality, as is common, acoustic receiver detection ranges vary substantially in this system, from 400 to 1500 m (Veilleux, 2014), so fish may be associating with any number of habitat features within 1 + km^2^ area. The only way to overcome this is to conduct studies with acoustic receiver arrays in tighter spacing to generate more precise fish positions. Regardless, characterizing habitat at a scale consistent with positioning precision is important to effectively characterize fish habitat with telemetry data.

Another key consideration is variation in acoustic receiver detection ranges over space and time, as variation in system performance may confound inferred patterns of fish habitat associations if not accounted for in analyses (Kessel et al., 2014; Kuai et al., 2021). Recently, a relatively simple and tractable approach has been developed (Brownscombe et al., 2020a), but was not integrated in the study design here. This is part of the reason why we elected to model fish habitat use at a species-level at a daily presence-absence scale, as opposed to total detections or residency, both of which are very likely to be impacted by variations in receiver performance. With this approach, a minimum of two detections by a single individual were required to generate a species-level presence on a given day. Although information is lost on the temporal extent and number of individuals occupying sites, this approach may help to buffer the effects of variation in receiver performance that were not well characterized, as well as the effects of tagging timing and the number of individuals being actively tracked over time. The latter was accounted for in our data filtering to some extent (i.e., filtering data to periods where > 5 individuals per species were being actively tracked; Appendix S1). However, for some species (i.e., yellow perch and bowfin) reliable tracking data was generated for < 1 year. It is not clear what duration of tracking is necessary to generate accurate assessments of fish habitat associations, which may also depend on species ecology, tracking system arrangement, and fish tagging numbers and locations. This should be the focus of future work.

In fish habitat management frameworks, the habitat suitability index (HSI) is a measure of the importance of habitat conditions or features in supporting fish populations and can take a variety of forms (de Kerckhove et al., 2008). We examined habitat associations at the species and daily presence/absence level, which could be interpreted as macrohabitat selection (type III HSI), with potential caveats discussed above, some of which we accounted for with analytical approaches within the study design. Therefore, we have interpreted the results as general spatial–temporal patterns in habitat associations. We were conservative given that likely some biotic and abiotic factors influencing fish habitat selection were not measured. Importantly, the habitats that fish occupied are not necessarily the most optimal for their productivity, as a variety of factors may result reduction in suitability (e.g., predators, habitat degradation, water quality, variable temperatures). Extra caution should be exercised when interpreting measures of habitat use as HSI in degraded habitats such as Toronto Harbour, where high quality habitat features may be very limited or non-existent. In these cases, habitat targets should be derived from data generated in more natural, healthy ecosystems that exist currently, or from historical data.

The study system here, Toronto Harbour, is subject to a variety of anthropogenic stressors associated with having the largest city in Canada in its riparian zone and extensive habitat modification, which has impacted the fish community (Hoyle et al., 2018). Indeed, all fish species examined here associated most with habitats in TTP and the Outer Harbour, which are less impacted by continual anthropogenic effects. Habitat restoration projects have been undertaken in some degraded regions in the Inner and Outer Harbour, although there is only limited evidence of their efficacy at attracting or supporting fishes (Rous et al., 2017; Veilleux et al., 2018). While extensive habitat enhancement efforts in TTP have recently been completed and a large habitat creation project at the mouth of the Don River is slated for completion by 2024 (Midwood et al., 2021a, b), their efficacy has yet to be assessed. Thus the measures of spatial–temporal habitat use generated here must be interpreted within this context—these are the habitats fish were occupying in a harbour that has been degraded by human development. Therefore, these are the habitats that continue to support these fish species, but may not necessarily be optimal, and may not be how they would use these habitats in a more pristine system. Nonetheless, habitat management may focus on protecting or further restoring habitats based on the current habitat’s importance, either at the community level (guided by the aggregate community level importance values generated here; Fig. 10), or on a species-specific basis using those model outputs (Figs. 3, 4, 5, 6, 7, 8 and 9). Indeed, focusing on sentinel species (e.g., predators such as northern pike, walleye, and largemouth bass that are ecologically important and also of recreational value) may be a straight-forward approach to set habitat goals that benefit a wide range of species. Habitat targets should be derived from the combination of this information as well as known habitat requirements of these species from less degraded habitats. Conducting telemetry studies to develop spatial–temporal habitat use patterns in more pristine habitats also would help to elucidate true habitat targets for these species and Toronto Harbour.

In summary, this study generated spatial–temporal habitat models for seven fish species that provide further insights into their ecology that should be valuable for fish habitat management in Toronto Harbour and similar systems and species. Each species exhibited unique spatial–temporal habitat associations that were generally consistent with their known ecology, and these associations would likely influence their responses to anthropogenic disturbance or habitat restoration. Toronto Harbour consists of a variety of fish habitat types that have been affected by a range of anthropogenic stressors (Doka et al. 2018), and the lesser-impaired outer harbour area is the most supportive of these fish species, with the majority using this region of the Harbour seasonally. TTP and Toronto Islands regions were generally the most occupied, especially for species like largemouth bass and bowfin that associate strongly with sheltered, high SAV habitat. These spatial–temporal patterns likely indicate habitat functions to some degree, with moderate-high SAV habitats supporting foraging and/or spawning for many species (i.e., largemouth bass, northern pike, bowfin, common carp), and low SAV habitats supporting others such as walleye and white sucker spawning in the spring season. Higher fish habitat degradation in the inner harbour area through infilling and shoreline hardening, water quality degradation, boat traffic, and/or environmental noise, are potential causes of more limited use by all species of this region, which has been subject to some, albeit limited, recent habitat restoration efforts (Rous et al., 2017; Veilleux et al., 2018). Beyond seasonal patterns, this study did not focus on variation in habitat associations over time, which continued telemetry monitoring and further data analysis may further elucidate. Informing fish habitat suitability indices is a relatively new application for acoustic telemetry data, and we discussed above some of the challenges and caveats with the current study design in doing so. Continued development of these approaches, including more extensive characterization of fish habitat at spatial–temporal scales consistent with telemetry data, as well as designing telemetry arrays and measuring and accounting for telemetry system performance (see Kessel et al. 2014), will make acoustic telemetry an increasingly useful tool for robust characterization fish habitat that integrates spatial and temporal domains and complements or tests prevailing methods.

## Supplementary Information

Below is the link to the electronic supplementary material.

Supplementary file1 (DOCX 4651 kb)

## Data Availability

The data supporting this work belong to numerous organizations and are currently being used for multiple scientific projects; hence, it is not being published alongside this article at this time. Data will be made available from the corresponding author on reasonable request.
