# Supplementary material for: Telemetry-based spatial–temporal fish habitat models for fishes in an urban freshwater harbour
Source: Hydrobiologia. 2023 Mar 16;850(8):1779–800. doi: 10.1007/s10750-023-05180-z (PMC10089985; doi:10.1007/s10750-023-05180-z)
Supplement: Supplementary file 1 — Supplementary file1 (DOCX 4651 kb) [file 10750_2023_5180_MOESM1_ESM.docx]

Table S1: Tracking metrics for seven fish species in Toronto Harbour using acoustic telemetry. Track start, end, and days tracked refers to the period of time where at least five individuals of the species were being actively tracked, and therefore data were included for analysis. Length indicates the mean ± standard deviation (minimum, maximum) amongst fish.

| *Species* | *Detections* | *ID count* | *Track start* | *Track end* | *Days tracked* | *Length (mm)* |
| --- | --- | --- | --- | --- | --- | --- |
| Bowfin | 355125 | 9 | 2013-07-12 | 2015-10-09 | 819 | 638±51 (562-725) |
| Common Carp | 4537984 | 81 | 2010-09-09 | 2019-10-30 | 3338 | 646±99 (340-854) |
| Largemouth Bass | 6673823 | 144 | 2010-09-22 | 2019-10-31 | 3326 | 400±93 (156-535) |
| Northern Pike | 12434510 | 155 | 2010-09-10 | 2019-10-28 | 3335 | 675±200 (250-1003) |
| Walleye | 595660 | 14 | 2012-05-17 | 2014-10-13 | 879 | 581±85 (423-703) |
| White Sucker | 2972831 | 33 | 2013-05-09 | 2019-10-25 | 2360 | 484±52 (388-562) |
| Yellow Perch | 342088 | 20 | 2012-09-12 | 2014-05-30 | 625 | 207±35 (158-271) |


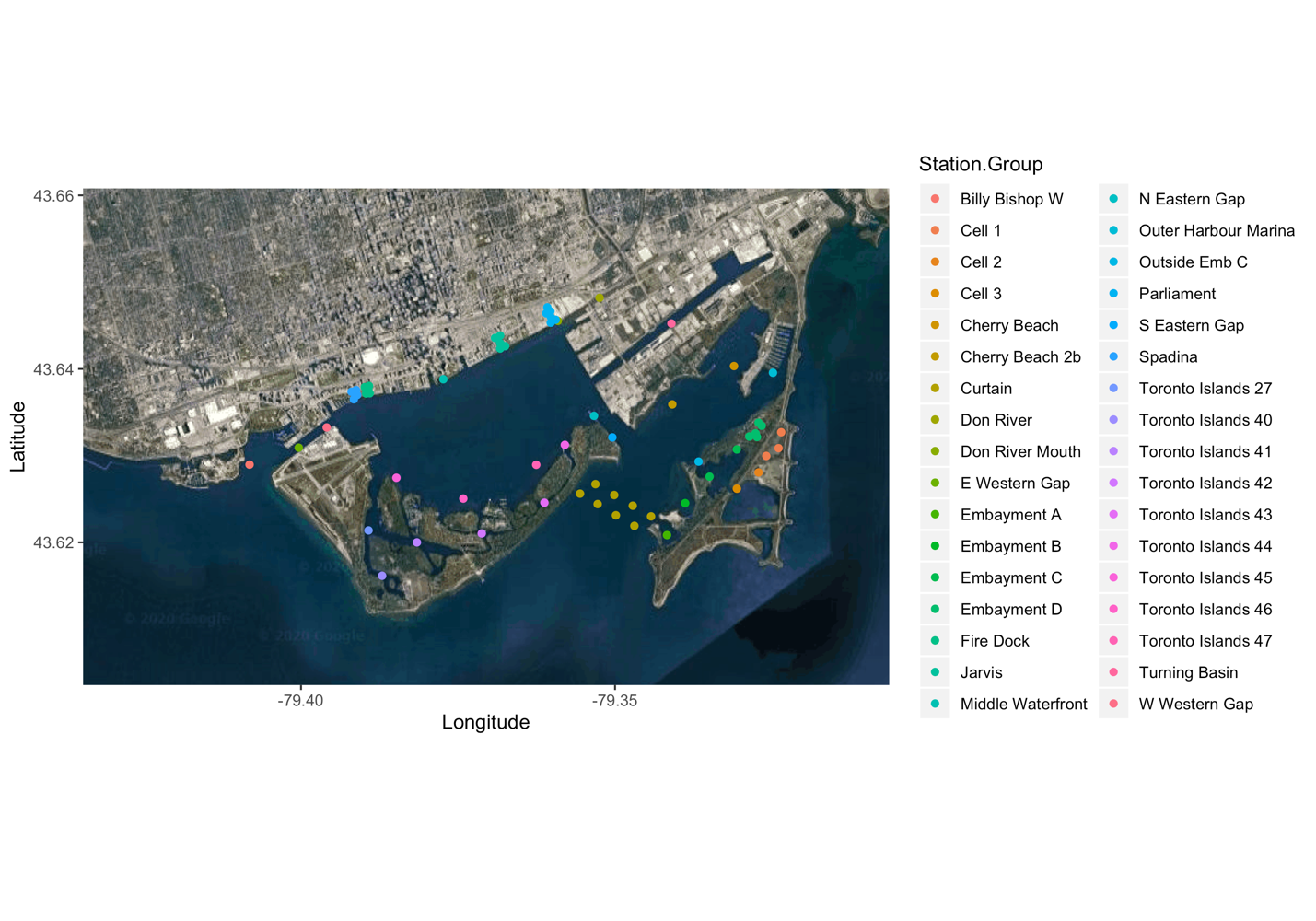


Fig. S1: Acoustic receiver deployment locations in Toronto Harbour


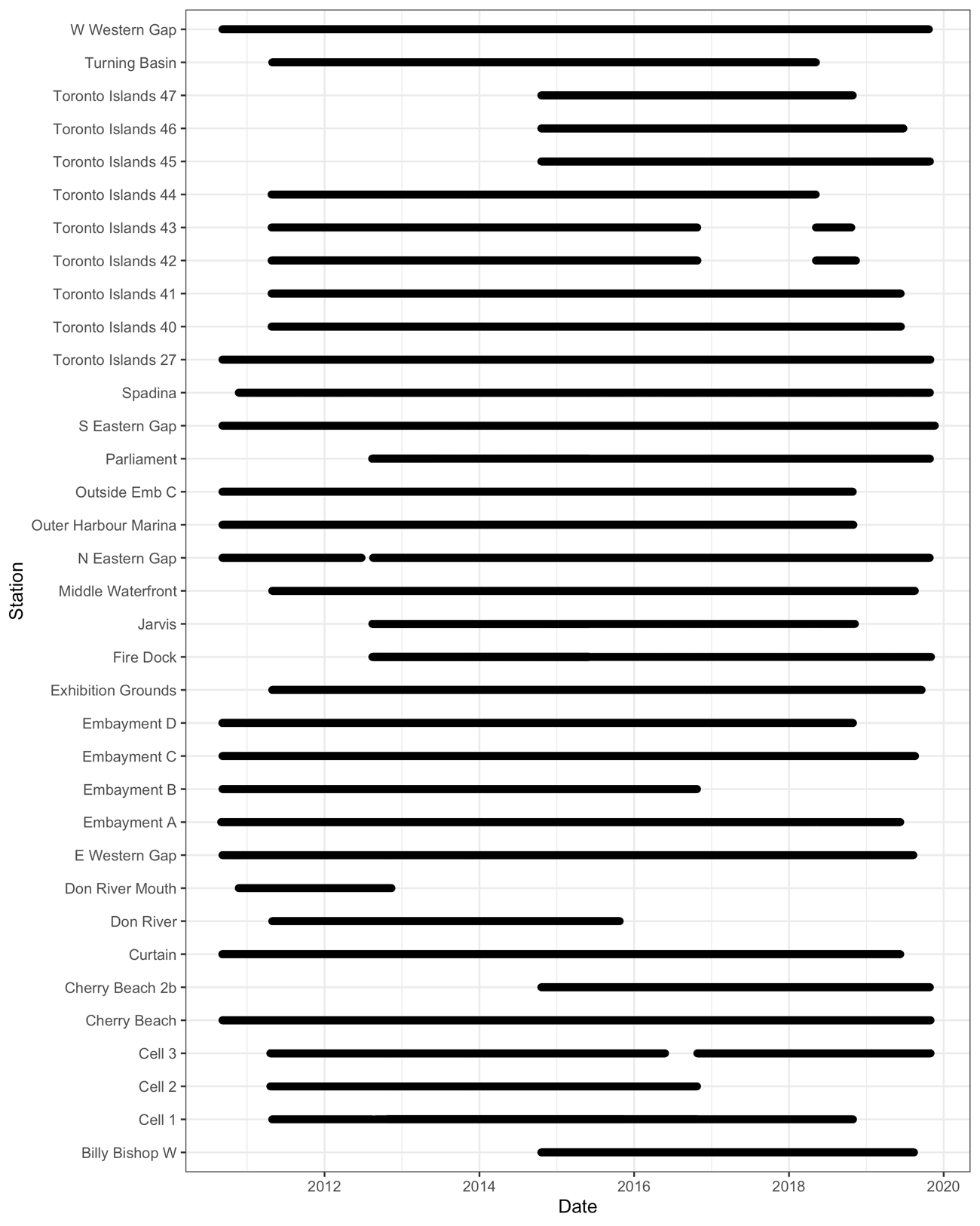


Fig. S2: Acoustic receiver deployment periods in Toronto Harbour


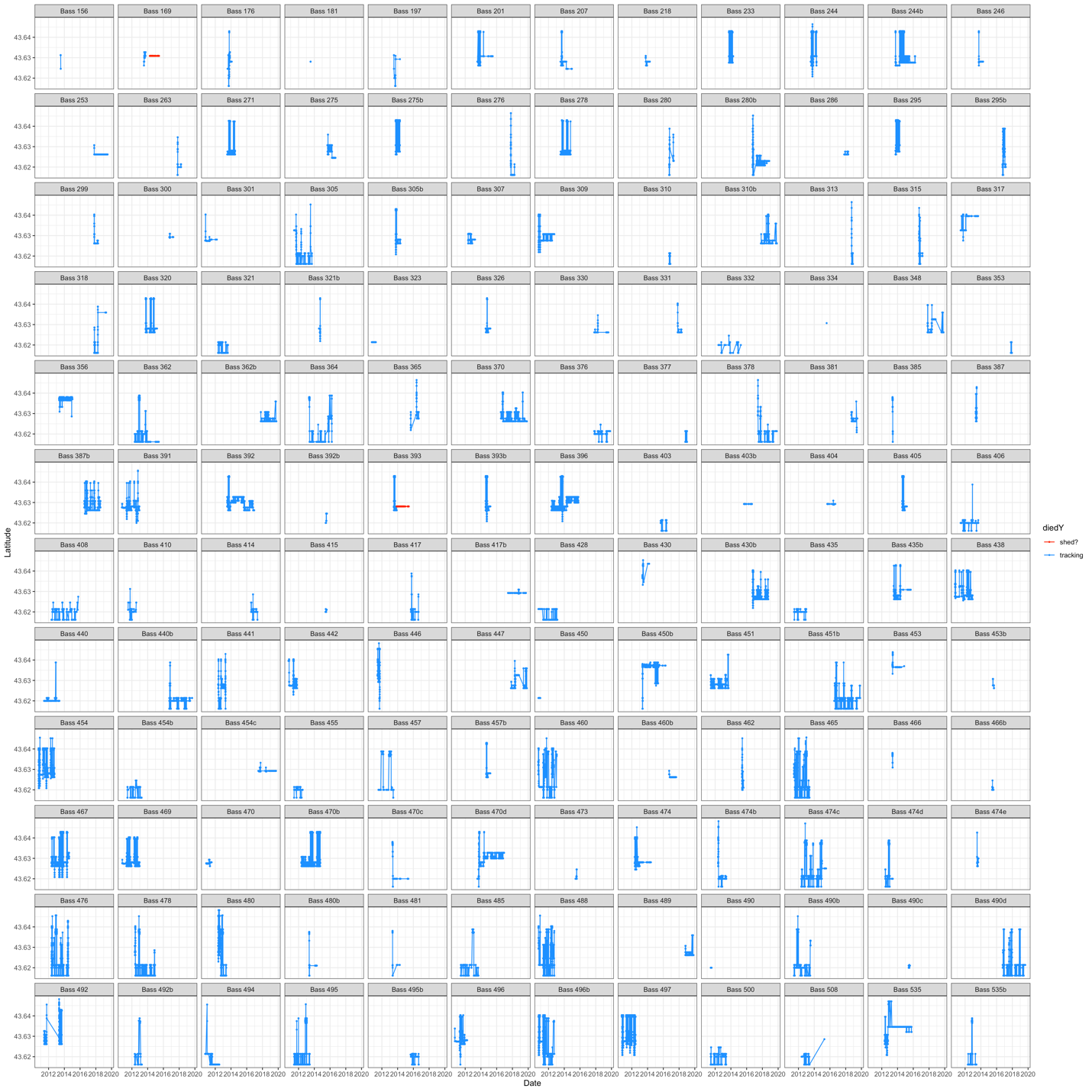


Fig. S3: Latitudinal space use of individual largemouth bass in Toronto Harbour over time. Periods where fish tracks were considered unreliable due to fish mortality or tag shedding are indicated in red, reliable tracks in blue. Unreliable tracks were considered as periods where the fish was detected repeatedly at the same acoustic receiver over long periods of time (multiple weeks+) and were not subsequently detected in other locations.


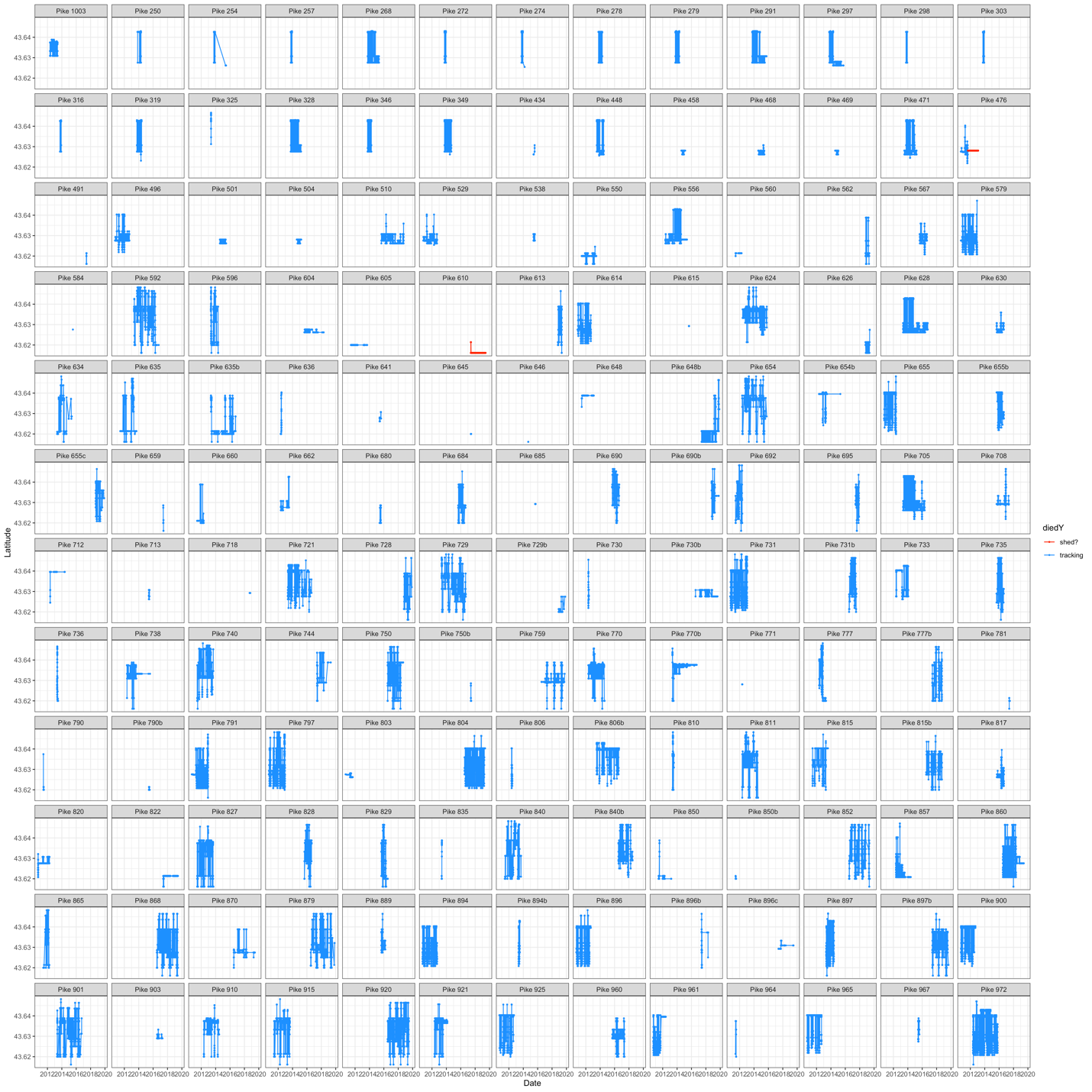


Fig. S4: Latitudinal space use of individual northern pike in Toronto Harbour over time. Periods where fish tracks were considered unreliable due to fish mortality or tag shedding are indicated in red, reliable tracks in blue. Unreliable tracks were considered as periods where the fish was detected repeatedly at the same acoustic receiver over long periods of time (multiple weeks+) and were not subsequently detected in other locations.


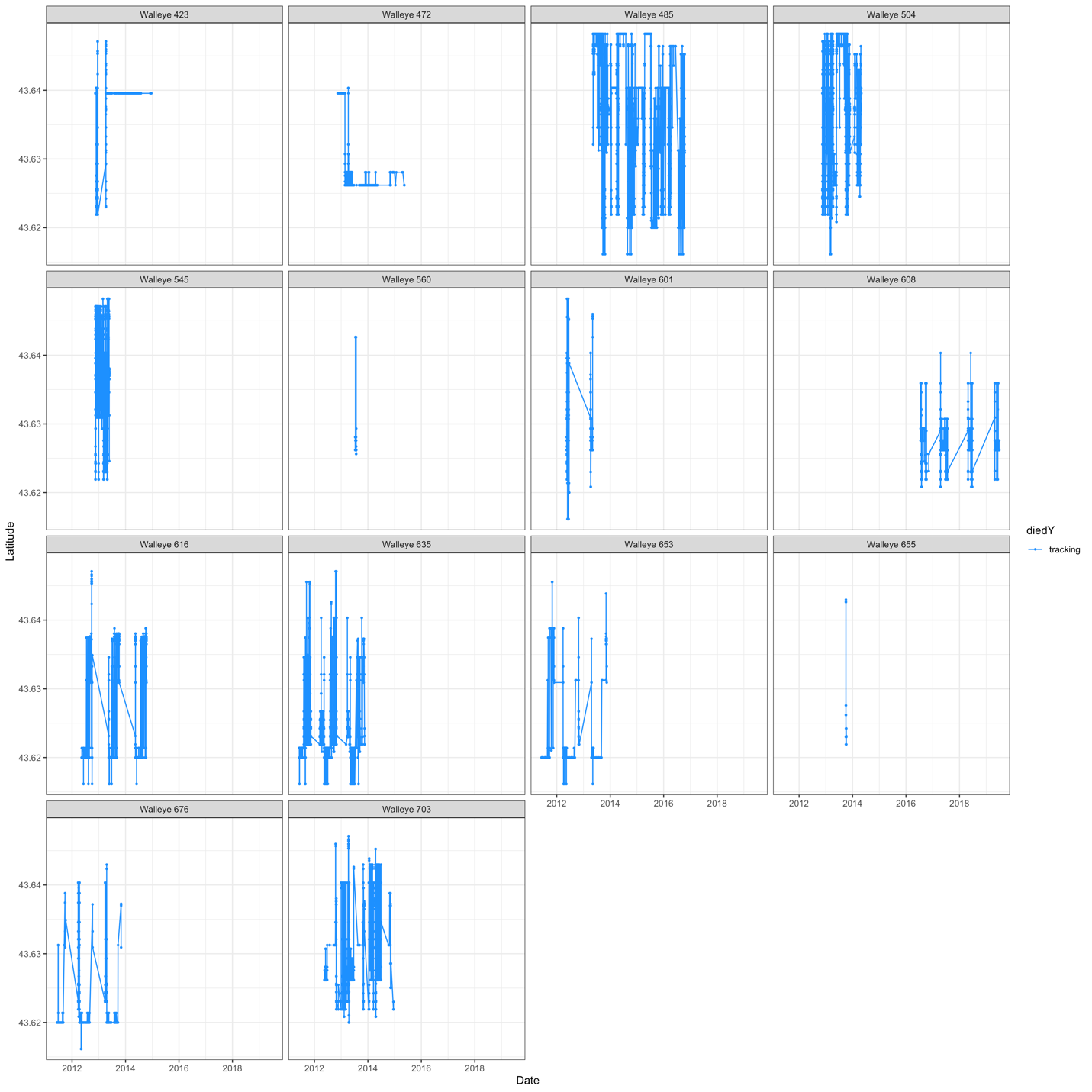


Fig. S5: Latitudinal space use of individual walleye in Toronto Harbour over time. Periods where fish tracks were considered unreliable due to fish mortality or tag shedding are indicated in red, reliable tracks in blue. Unreliable tracks were considered as periods where the fish was detected repeatedly at the same acoustic receiver over long periods of time (multiple weeks+) and were not subsequently detected in other locations.


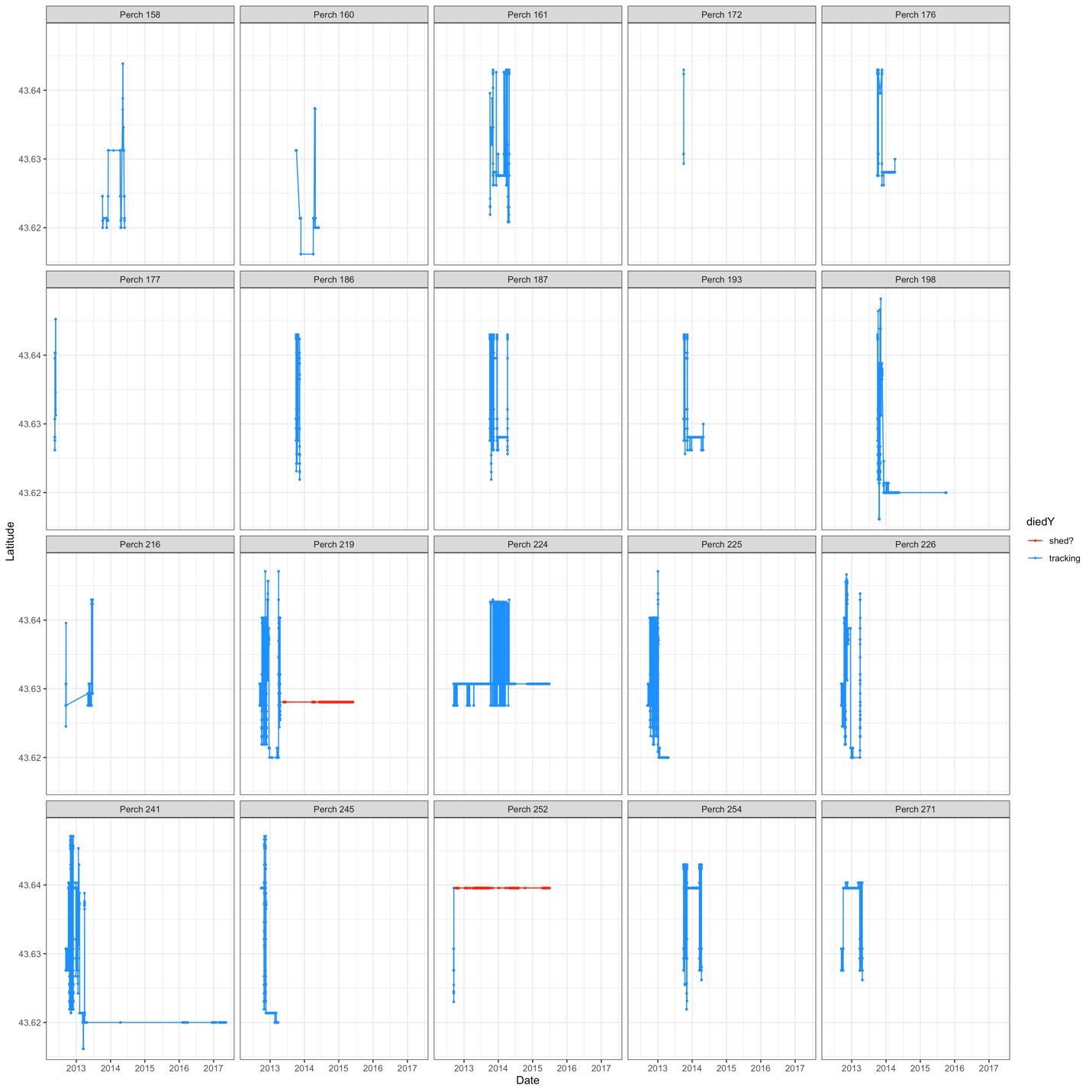


Fig. S6: Latitudinal space use of individual yellow perch in Toronto Harbour over time. Periods where fish tracks were considered unreliable due to fish mortality or tag shedding are indicated in red, reliable tracks in blue. Unreliable tracks were considered as periods where the fish was detected repeatedly at the same acoustic receiver over long periods of time (multiple weeks+) and were not subsequently detected in other locations.


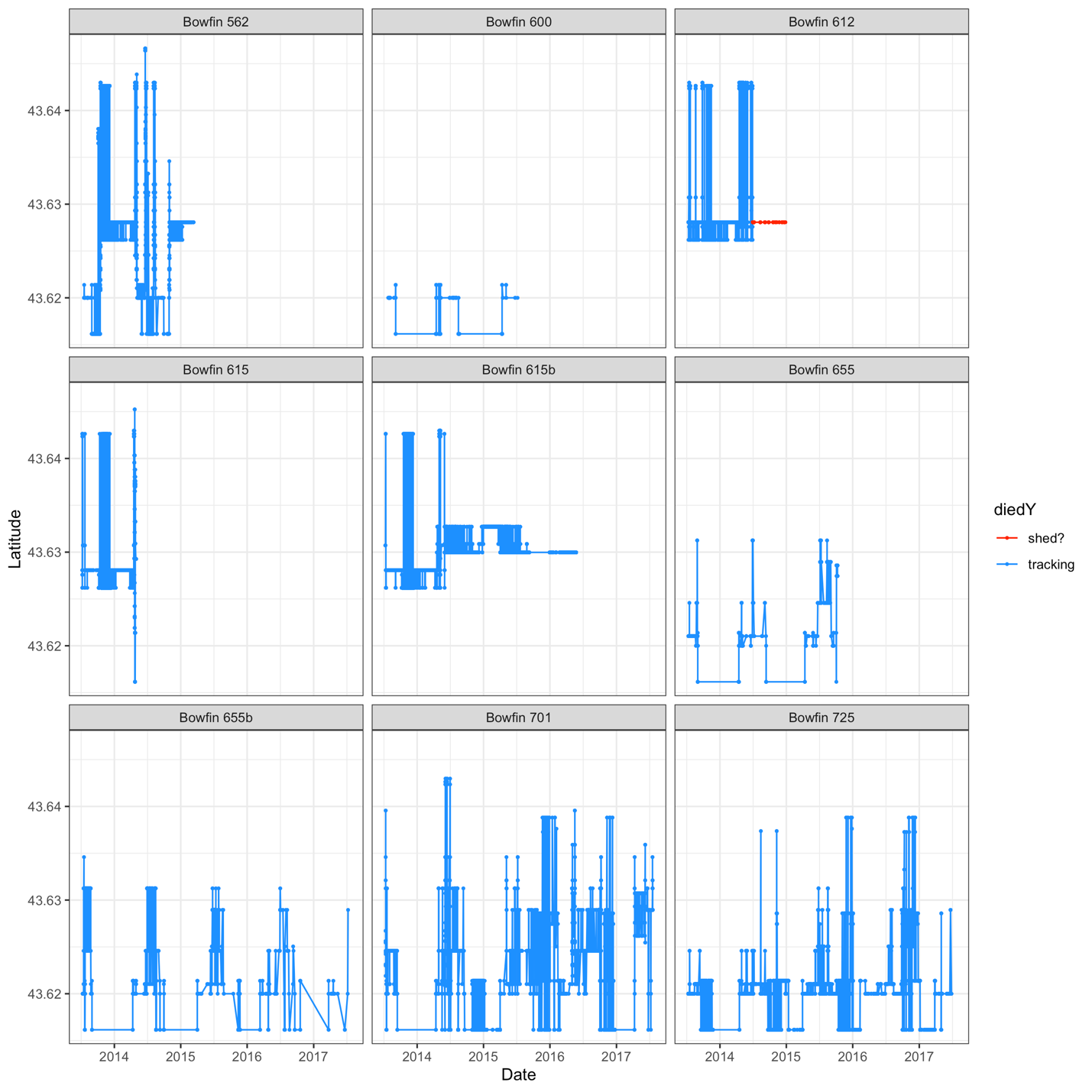


Fig. S7: Latitudinal space use of individual bowfin in Toronto Harbour over time. Periods where fish tracks were considered unreliable due to fish mortality or tag shedding are indicated in red, reliable tracks in blue. Unreliable tracks were considered as periods where the fish was detected repeatedly at the same acoustic receiver over long periods of time (multiple weeks+) and were not subsequently detected in other locations.


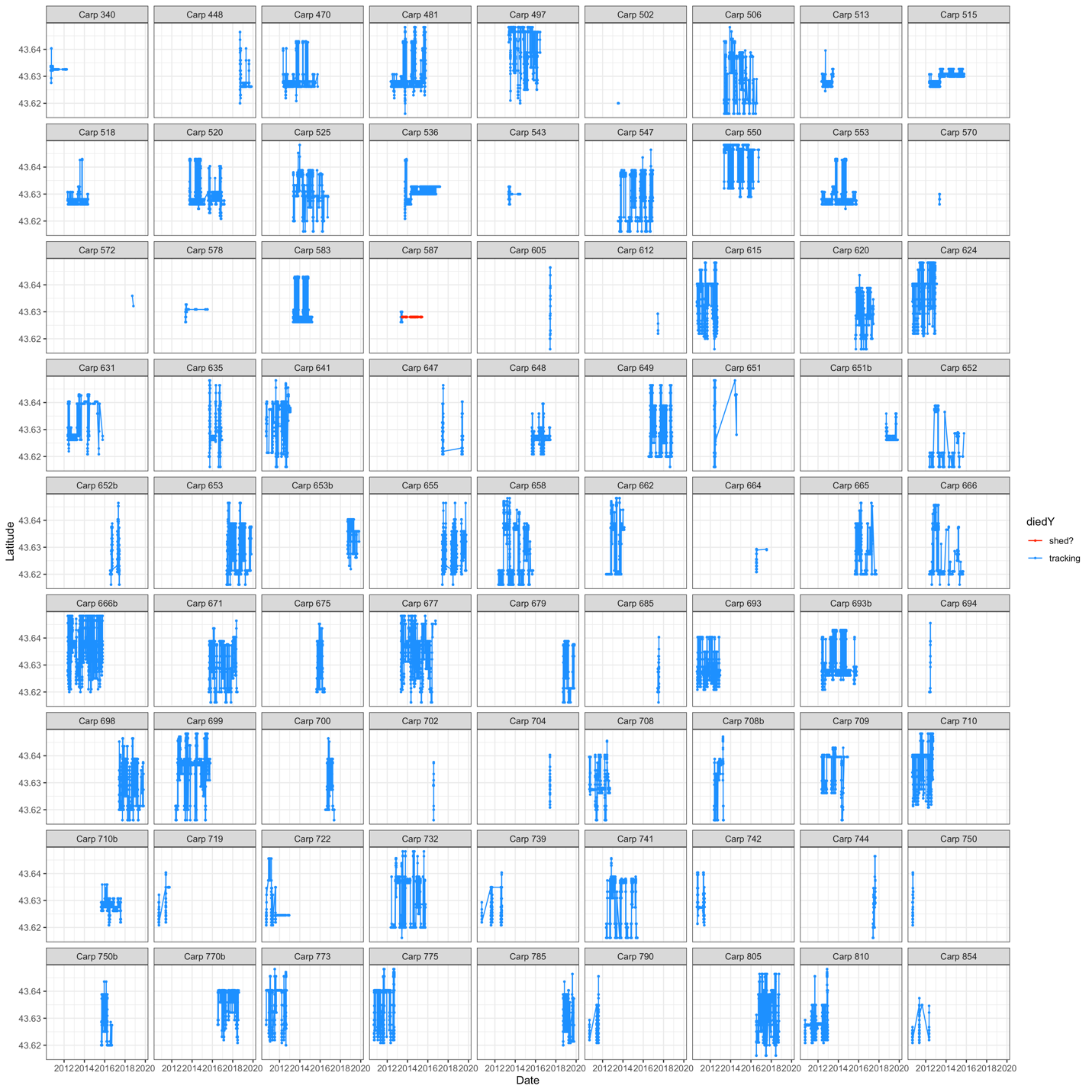


Fig. S8: Latitudinal space use of individual common carp in Toronto Harbour over time. Periods where fish tracks were considered unreliable due to fish mortality or tag shedding are indicated in red, reliable tracks in blue. Unreliable tracks were considered as periods where the fish was detected repeatedly at the same acoustic receiver over long periods of time (multiple weeks+) and were not subsequently detected in other locations.


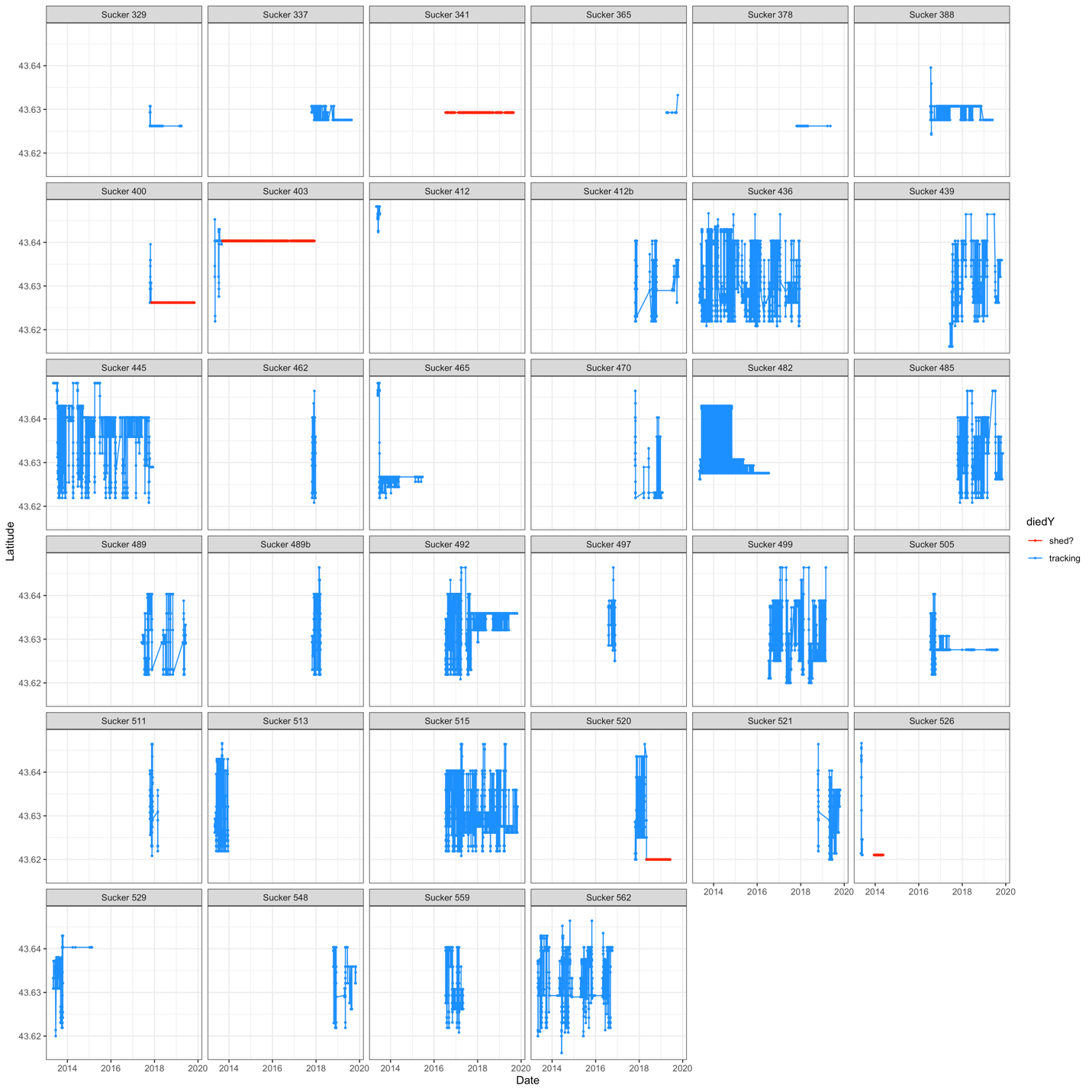


Fig. S9: Latitudinal space use of individual white sucker in Toronto Harbour over time. Periods where fish tracks were considered unreliable due to fish mortality or tag shedding are indicated in red, reliable tracks in blue. Unreliable tracks were considered as periods where the fish was detected repeatedly at the same acoustic receiver over long periods of time (multiple weeks+) and were not subsequently detected in other locations.


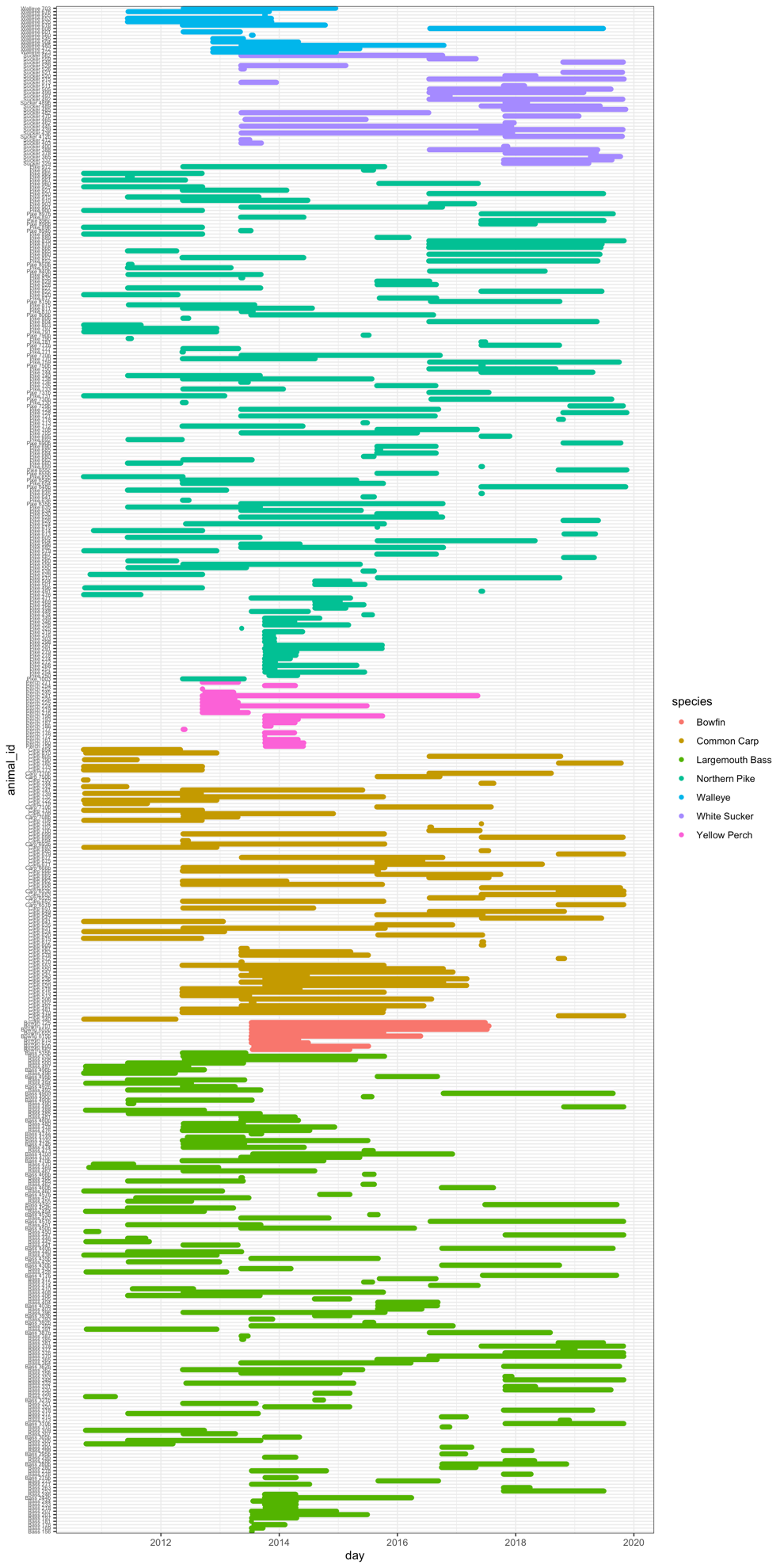


Fig. S10: Abacus plot of fish tracking periods in Toronto Harbour


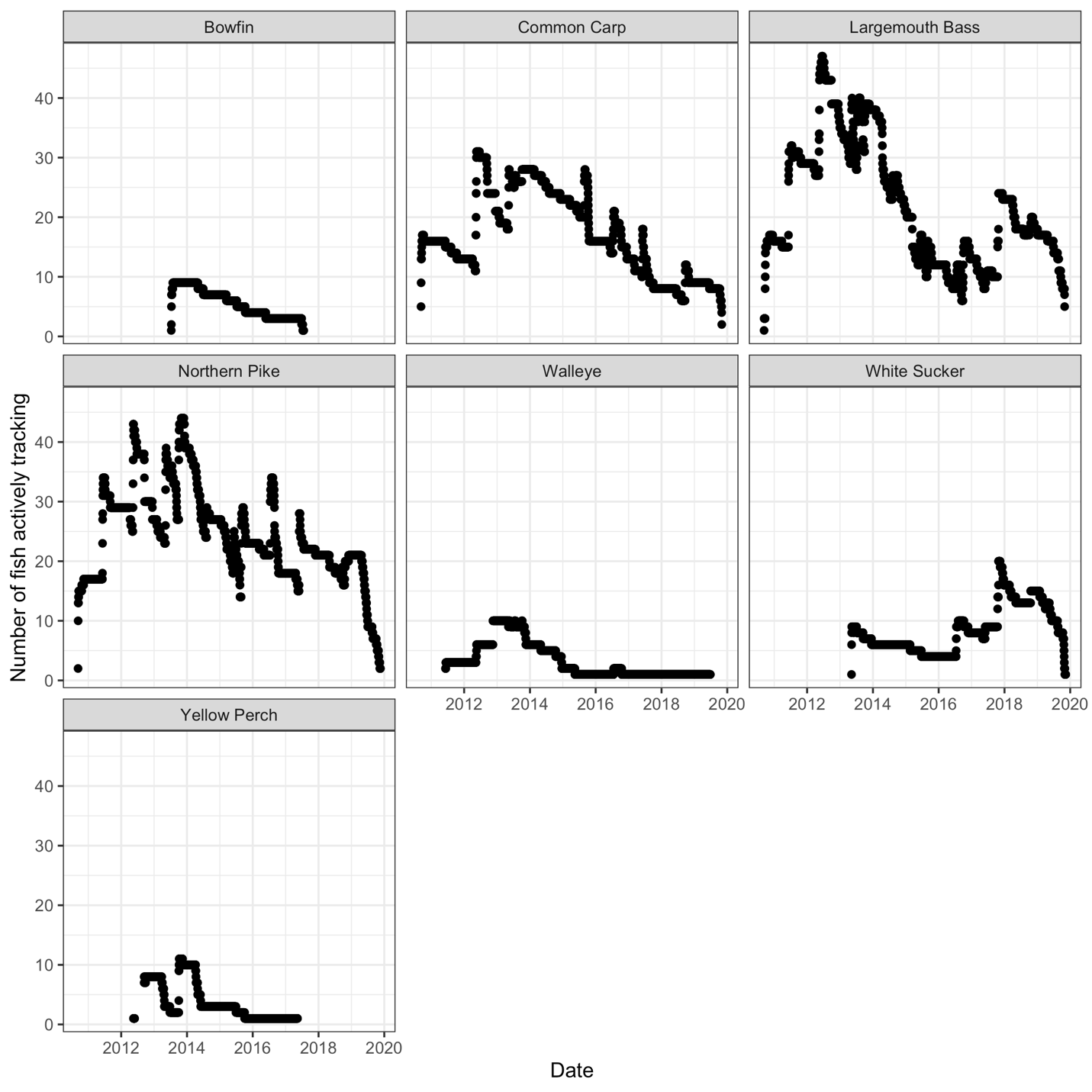


Fig. S11: The number of individuals of each fish species that were tracked with acoustic telemetry over the study period. Only periods where at least five individuals were being tracked were included in further analysis.
